# Tumor- and cytokine-primed human natural killer cells exhibit distinct phenotypic and transcriptional signatures

**DOI:** 10.1371/journal.pone.0218674

**Published:** 2019-06-26

**Authors:** May Sabry, Agnieszka Zubiak, Simon P. Hood, Poppy Simmonds, Helena Arellano-Ballestero, Eily Cournoyer, Meghavi Mashar, A. Graham Pockley, Mark W. Lowdell

**Affiliations:** 1 Department of Haematology, University College London, London, United Kingdom; 2 John van Geest Cancer Research Centre, Nottingham Trent University, Nottingham, United Kingdom; 3 InmuneBio Inc., La Jolla, California, United States of America; Universita degli Studi di Palermo, ITALY

## Abstract

An emerging cellular immunotherapy for cancer is based on the cytolytic activity of natural killer (NK) cells against a wide range of tumors. Although *in vitro* activation, or “priming,” of NK cells by exposure to pro-inflammatory cytokines, such as interleukin (IL)-2, has been extensively studied, the biological consequences of NK cell activation in response to target cell interactions have not been thoroughly characterized. We investigated the consequences of co-incubation with K562, CTV-1, Daudi RPMI-8226, and MCF-7 tumor cell lines on the phenotype, cytokine expression profile, and transcriptome of human NK cells. We observe the downregulation of several activation receptors including CD16, CD62L, C-X-C chemokine receptor (CXCR)-4, natural killer group 2 member D (NKG2D), DNAX accessory molecule (DNAM)-1, and NKp46 following tumor-priming. Although this NK cell phenotype is typically associated with NK cell dysfunction in cancer, we reveal the upregulation of NK cell activation markers, such as CD69 and CD25; secretion of pro-inflammatory cytokines, including macrophage inflammatory proteins (MIP-1) α /β and IL-1β/6/8; and overexpression of numerous genes associated with enhanced NK cell cytotoxicity and immunomodulatory functions, such as *FAS*, *TNFSF10*, *MAPK11*, *TNF*, and *IFNG*. Thus, it appears that tumor-mediated ligation of receptors on NK cells may induce a primed state which may or may not lead to full triggering of the lytic or cytokine secreting machinery. Key signaling molecules exclusively affected by tumor-priming include *MAP2K3*, *MARCKSL1*, *STAT5A*, and *TNFAIP3*, which are specifically associated with NK cell cytotoxicity against tumor targets. Collectively, these findings help define the phenotypic and transcriptional signature of NK cells following their encounters with tumor cells, independent of cytokine stimulation, and provide insight into tumor-specific NK cell responses to inform the transition toward harnessing the therapeutic potential of NK cells in cancer.

## Introduction

Natural killer (NK) cell responses are influenced by signals from numerous germline-encoded receptors on the cell surface. NK cell-mediated cytotoxicity against target tumor cells requires the co-engagement of several activation receptors—such as the natural cytotoxicity receptors (NCRs) NKp46 and NKp80—or other co-stimulatory receptors—including natural killer group 2 member D (NKG2D) or DNAX accessory molecule (DNAM)-1 [1]. Once activated, NK cells exhibit cytotoxicity against a variety of neoplastic cells and produce a wide array of immunoregulatory cytokines, such as interferon (IFN)-γ and tumor necrosis factor (TNF)-α [2]. Activated NK cells can also secrete chemokines that aid in initiating inflammatory responses and recruiting other immune cells to inflammatory sites, including macrophage inflammatory proteins (MIP)-1α/β, IL-8, and RANTES (resting and normal T cell expressed and secreted).

*In vitro* activation of NK cells can be achieved via target cell recognition and/or exposure to one or more pro-inflammatory cytokines. Pre-incubating NK cells with IL-2 results in the generation of a lymphokine-activated killer (LAK) cell, whereas pre-incubating with target tumor cells generates a “tumor-primed” NK cell (TpNK) [3, 4]. In both cases, the “primed” state can be defined by an NK cell’s ability to kill target cells that previously exhibited little to no sensitivity to resting NK cells. Although cytokines that share a common cytokine-receptor gamma chain (or CD132) such as IL-2 and IL-15 exhibit some redundant signaling, they also have specific functions in regulating NK cell responses [5]. The gamma-chain receptor associates with the Janus tyrosine-kinase (JAK)-3 to phosphorylate different downstream signal transducer and activator of transcription (STAT) molecules depending on the type of receptors engaged. Exposure to these cytokines alone or in combination with others, such as IL-12 or IL-18, can induce different and, in some cases synergistic, effects on NK cell effector functions. This cooperation can also be achieved via non-redundant ligand combinations in target cells.

Human NK cell responses to exogenous cytokines have been extensively studied; however, relatively little is known about the biological and functional consequences of NK cell priming following interaction with target cells. Previous studies on the activation profile of cytokine-stimulated NK cells have revealed that NK cells upregulate receptors and key molecules involved in the cell cycle, cell proliferation, and immune responses [6]. Although different effects have been reported depending on the cytokines used, the majority of altered NK cell transcripts are involved in cytokine/chemokine signaling. In contrast, studies characterizing the tumor-associated NK cell profile using NK cells isolated from cancer patients have reported downregulation of NK cell activation receptors, and genes involved in cytokine signaling [7, 8]. Although the downregulation of NK cell activation receptors is typically associated with decreased NK cell activity and increased disease severity, it has recently been reported to be an important mechanism for NK cell survival, motility, and serial engagement of target cells [9]. *In vitro* models of NK cell responses to tumors have focused on the prototypical NK cell-sensitive leukemic cell line, K562 [10]. We have previously demonstrated that another leukemic cell line, CTV-1, is capable of priming NK cells to display enhanced effector functions [3]. Although both K562 and CTV-1 cells are sensitive to NK cell killing, CTV-1 cells are less susceptible and can “prime” NK cells for subsequent killing of targets [4]. In contrast, it has been reported that the encounter with K562 cells induces functional anergy in NK cells and renders them less responsive to further stimulation [11–13].

We investigated the consequences of tumor cell- or cytokine-based priming on the phenotype, cytokine secretion profile, and transcriptome of NK cells to identify signatures that differentiate these cell populations. In summary, priming human NK cells from healthy individuals with tumor cells downregulates the expression of activation receptors, triggers the secretion of pro-inflammatory cytokines, and induces differential gene expression, which is tumor cell-dependent and distinct to that induced by lymphokine activation. Understanding tumor-specific NK cell responses is an important step toward maximizing the therapeutic potential of NK cells in cancer treatment.

## Materials and methods

### Cells

Peripheral blood (PB) samples were obtained from healthy donors with prior informed consent. Peripheral blood mononuclear cells (PBMCs) were obtained by density gradient centrifugation from which NK cells were isolated using positive selection for CD56 (CD56 MicroBeads; Miltenyi Biotec) or negative selection using EasySep Human NK Cell Enrichment Kit (StemCell Technologies). Both procedures were conducted according to the manufacturers’ protocols with purity routinely >92% CD56^+^CD3^-^. Freshly-isolated NK cells were maintained in complete growth medium (CM) (RPMI 1640 supplemented with 10% v/v fetal bovine serum and 2mM l-glutamine; all from Life Technologies). The K562 chronic myelogenous leukemia cell line, CTV-1 acute lymphoblastic leukemia cell line, Daudi Burkitt lymphoma cell line, RPMI-8226 myeloma cell line and MCF-7 breast cancer cell line (DSMZ) were maintained in CM. NK cell stimulation was achieved by co-incubating CD56^+^CD3^-^ NK cells (1x10^6^) with untreated or mitomycin-C-treated K562, CTV-1, Daudi, RPMI-8226 or MCF-7 cells (2x10^6^), or the exogenous cytokines IL-2 (200 IU/ml), IL-12 (20 ng/ml), IL-15 (10 ng/ml) or IL-18 (100 ng/ml) (R&D Systems) alone or in combination as indicated, for 6 or 16 hours at 37°C, 5% v/v CO_2_ in CM. Obtaining blood from healthy donors was approved by the University College London-Royal Free Hospital Biobank Ethical Review Committee (NC.2015.019).

### Flow cytometry

The following fluorochrome-conjugated monoclonal antibodies (mAbs) were purchased from BD Biosciences: CD56 (clone B159), CD3 (clone SK7), CD314 (NKG2D; clone 1D11), CD226 (DNAM-1; clone DX11), CD25 (clone M-A251), CD62L (clone DREG-56), CD69 (clone L78), CD184 (CXCR4; clone 12G5), CD335 (NKp46; clone 9E2), and CD337 (NKp30; clone p30-15). CD336 (Nkp44; clone 253415), STAT5A (clone 251610), VEGF (clone 23410), ULBP1 (clone 170818), ULBP3 (clone 166510), ULBP2/5/6 (clone 165903), and B7H6 (clone 875001) mAbs were purchased from R&D Systems. NKp80 (clone 4A4.D10), CD16 (clone VEP3), CD137 (4-1BB; clone 4b4-1), IFN-γ (clone 45–15), TNF-α (clone cA2), MIP-1α (CCL3; clone REA257), MIP-1β (CCL4; clone REA511), MICA/B (clone 6D4), CD155 (clone PV404.19), RANTES (CCL5; clone REA346), and CD244 (2B4; clone REA112) mAbs were purchased from Miltenyi Biotech. CD11a (clone HI111), CD54 (clone RR1/1), and CD57 (clone TB01) mAbs were purchased from eBiosciences. CD181 (CXCR1; clone 8F1/CXCR1), CXCR7 (clone 8F11-M16), CD112 (clone TX31) and HLA-A/B/C (clone W6/32) mAbs were purchased from Biolegend. TNFAIP3 (A20; clone EPR2663), and MAP2K3 (clone EPR17345-104) mAbs were purchased from Abcam.

To analyze cell surface antigen expression, cells (10^5^ cells in 100 μl of Hank’s Balanced Salt Solution, HBSS) were incubated with fluorochrome-conjugated mAbs at the manufacturer’s recommended concentration for 15 minutes at room temperature. For intracellular staining, after surface staining, cells were washed, fixed, permeabilized (Cytofix/Cytoperm; Becton Dickinson), and stained with fluorochrome conjugated mAbs. Finally, cells were washed and analyzed using flow cytometry (MACSQuant with MACSQuantify software; Miltenyi Biotec or Novocyte with NovoExpress software; ACEA Biosciences). Brefeldin A (GolgiPlug; Becton Dickinson) and Monensin (GolgiStop; Becton Dickinson) were added 5 hours before the end of the incubation period. The applicable isotype and fluorescence minus one (FMO) controls were included in each experiment. A minimum of 20,000 CD56^+^CD3^-^ events were acquired after electronic gating on viable cells using forward (FSC) and side (SSC) scatter signals and TO-PRO-3 iodide staining (Thermo Fisher Scientific).

### Cytokine secretion measurements

Freshly-isolated resting NK cells (2×10^5^) were washed twice and incubated alone or with tumor cell lines (4×10^5^), IL-2 (200 IU/ml), or IL-12 (20 ng/ml) in 200 μL of CM for the indicated time at 37°C in 5% v/v CO_2_. Thereafter, cell-free supernatants were collected and stored at −80°C pending analysis using a 25-plex immunoassay (Luminex 100 IS; Invitrogen). Values from blank controls, or blank controls with target cells incubated alone as in the case of target-stimulated NK cells, were subtracted from the data. To quantify human transforming growth factor beta (TGF-β) secretion, supernatants were analyzed using a Quantikine enzyme-linked immunosorbent assay (ELISA) kit (R&D Systems) according to the manufacturer’s instructions. Optical density values from standard controls were used to interpolate X values of secretion measurements in pg/ml following baseline correction using blank controls.

### RNA-sequencing

The transcriptome of NK cells after stimulation with tumor cells or cytokines was profiled by RNA-sequencing (RNA-Seq). For this, resting NK cells (5×10^5^) were washed twice and incubated alone or with either mitomycin-C-treated K562 or CTV-1 cells (1x10^6^) for 6 hours or with IL-2 (200 IU/ml) for 16 hours at 37°C in 5% v/v CO_2_, after which NK cells were isolated from the co-culture using positive selection for CD56. RNA was extracted from the samples using the RNAqueous Micro Kit (Thermo Fisher Scientific) and stored at −80°C. To prepare the library, 100 ng of total RNA was processed using the KAPA mRNA HyperPrep Kit (p/n KK8580) according to the manufacturer’s instructions. Briefly, mRNA was isolated from total RNA using Oligo dT beads to pull down poly-adenylated transcripts. The purified mRNA was fragmented using chemical hydrolysis (heat and divalent metal cation) and primed with random hexamers. Strand-specific, first strand cDNA was generated via a reverse transcriptase procedure in the presence of Actinomycin D. This allows for RNA-dependent synthesis while preventing spurious DNA-dependent synthesis. The second cDNA strand was synthesized using dUTP in place of dTTP to mark the second strand. The resultant cDNA was then “A-tailed” at the 3’ end to prevent self-ligation and adapter dimerization. Full-length xGen adaptors (IDT) containing two unique 8-bp sample-specific indexes, a unique molecular identifier (N8), and a T overhang were ligated to the A-Tailed cDNA. Successfully ligated cDNA molecules were then enriched with limited cycle PCR (14 cycles; the actual number is dependent on the amount of input RNA). The high-fidelity polymerase employed in the PCR is unable to extend through uracil. Thus, only first strand cDNA was amplified for sequencing, making the library strand-specific (first-strand). Prior to sequencing, libraries to be multiplexed in the same run were pooled in equimolar quantities, calculated from Qubit and Bioanalyser fragment analysis. Samples were sequenced on the NextSeq 500 instrument (Illumina) using a 75 bp single read run with a corresponding 8 bp UMI read. For data analysis, run data were demultiplexed and converted to fastq files using Illumina’s bcl2fastq Conversion Software v2.19. Fastq files were then aligned to the human genome UCSC hg38 using RNA-STAR 2.5.2b then UMI deduplicated using Je-suite (1.2.1). Reads per transcript were counted using FeatureCounts and differential expression was estimated using the BioConductor package SARTools, which is a DESeq2 wrapper. All annotations and sequences were obtained from Illumina iGenomes (http://emea.support.illumina.com/sequencing/sequencing_software/igenome.html). Functional annotation analysis using gene ontology (GO) terms was performed on gene data sets that met the cutoff values (>1 Log2fold change, P-value <0.05) using the Panther Classification System gene list analysis tool (http://pantherdb.org)

### NanoString nCounter gene expression assay

Additional gene expression profiling was conducted using a NanoString Technologies nCounter FLEX amplification-free gene expression profiling platform. The nCounter system directly detects and counts nucleic acids via reporter probes appended with multiple fluorophore barcodes and biotinylated capture probes that attach to microscopic beads. These are affixed to lanes in a translucent cartridge and read in an optical scanner. To prepare the samples, resting NK cells (5×10^5^) were washed twice and incubated alone or with mitomycin-C-treated CTV-1 cells (1×10^6^) for 6 hours or with IL-2 (200 IU/ml) for 16 hours at 37°C in 5% v/v CO_2_. Thereafter, NK cells were selectively enriched from co-culturing by CD56 isolation, and RNA was extracted from the samples using the RNAqueous Micro Kit (Thermo Fisher Scientific). Batches of 12 separate samples at one time were prepared according to the manufacturer’s instructions and hybridized with probes at 65°C for 18 hours. They were then placed into the automated nCounter Prep Station (NanoString Technologies) in which samples were affixed to cartridges. Cartridges were immediately placed into the nCounter Digital Analyzer (NanoString Technologies) and read at maximum resolution. Gene expression was directly measured through counts of corresponding mRNA in each sample using nSolver software (NanoString Technologies). Following background thresholding and normalization using the geometric mean of positive controls and housekeeping genes, differential expression was calculated against NK alone control samples as a baseline.

### Ingenuity pathway analysis

Functional analysis of gene expression changes was undertaken using Ingenuity Pathway Analysis (IPA, Ingenuity Systems). This software analyzes RNA expression data in the context of known biological responses, regulatory networks, and other higher-order response pathways. IPA identified canonical pathways, upstream regulators, signaling networks, biological functions, and/or diseases that were most significant. Genes from the data set that met the cutoff values (>1 Log2fold change, P-value <0.05) were considered for analysis. The Fisher's exact test was used for all analyses to calculate a P-value determining the probability that each biological function assigned to the data set was due to chance alone. The overall activation/inhibition states are predicted based on a Z-score algorithm, which is the negative log of the P-value derived from the Fisher’s exact test.

### Statistical analysis

Antigen expression and cytokine secretion profiles were analyzed with GraphPad software (GraphPad Software). Statistical comparisons between normally distributed pairs of matched samples (confirmed by the F test) were tested for significant differences in their means by the paired *t*-test. Variables are presented as means ± standard deviation (SD) or as a percentage change in mean relative to untreated samples (% change in mean ± SD of difference). P-values <0.05 were considered statistically significant.

## Results

### Tumor-priming results in a loss of NK cell activation receptor expression

**Fig 1:**
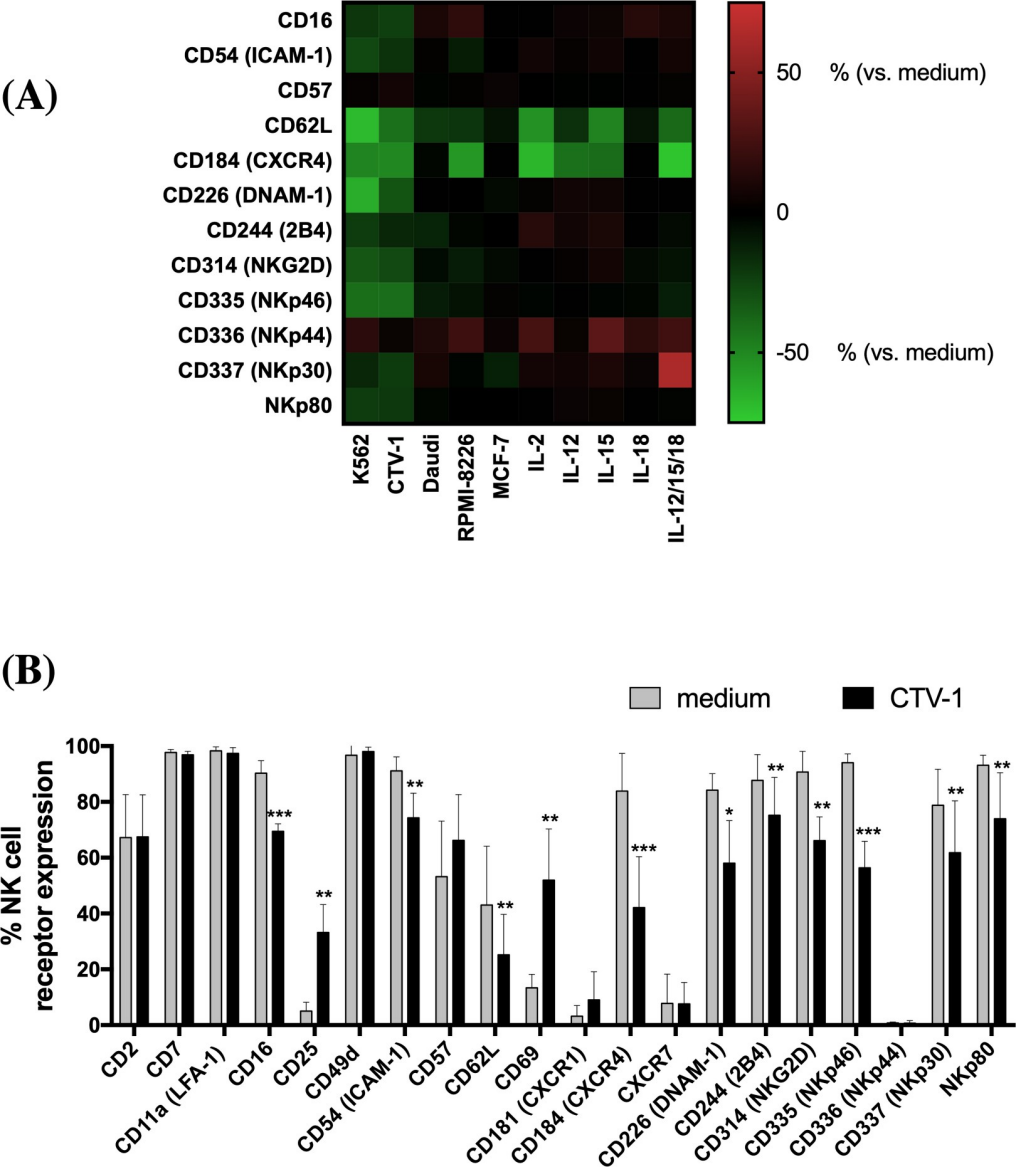
Loss of NK cell activation receptor expression following tumor-priming. (A) Freshly isolated NK cells were incubated in medium alone, with K562, CTV-1, Daudi, RPMI-8226, or MCF-7 cells or with IL-2/12/15/18 (individually or in combination) overnight at 37°C. Thereafter, cells were washed. NK cell activation receptor expression was analyzed using flow cytometry. The percentage change of NK cell expression was calculated for each receptor after stimulation and results are presented as the mean of 3–5 different donors. Red indicates an increase, and green indicates a decrease in receptor expression, according to the key on the right, relative to NK cells in medium alone. (B) Freshly isolated NK cells were incubated in medium alone or with CTV-1 cells overnight at 37°C. Cells were washed, the surface expression of 20 different NK cell activation receptors was analyzed. Bars represent the means ± SD of 5 different donors. The percentages of NK cell expression of different receptors after stimulation was compared with those in cells treated with medium alone using the paired *t-*test. Statistical significance is indicated as: *P <0.05; **P <0.01; ***P <0.001.

Loss of NK cell activation receptor expression is commonly reported in cancer [14–16]. We set out to determine the antigen expression profiles of peripheral blood-derived NK cells from healthy donors upon exposure to tumor targets or cytokines by incubating freshly-isolated cells in medium alone, with K562, CTV-1, Daudi, RPMI-8226, or MCF-7 tumor cell lines, the individual cytokines IL-2, IL-12, IL-15, or IL-18, or a combination of IL-12/15/18. Cells were washed, and the expression of NK cell activation receptors was determined by flow cytometry (**Fig 1A)**. As expected, NK cells exhibited similar or higher expression of the senescence marker CD57 and the activation marker NKp44 following stimulation with different tumor cell lines or cytokines. In line with previous studies, co-incubation of NK cells with the leukemic cell line K562 significantly reduced the number of NK cells expressing the Fc receptor CD16 and the lymph node trafficking receptor CD62L compared to NK cells incubated in medium alone (p < 0.01) [17]. NK cell stimulation using K562 also significantly decreased the percentage of NK cells expressing the bone marrow trafficking receptor C-X-C chemokine receptor (CXCR)-4; the co-stimulatory receptors intracellular adhesion molecule (ICAM)-1, DNAM-1, 2B4 and NKG2D; and the NCRs NKp46 and NKp80 (p < 0.04).

K562 is sensitive to NK cells due to low to no expression of MHC class I molecules and the expression of ligands for NK cell co-stimulatory receptors such as NKG2D, DNAM-1, NKp30, CD2, and LFA-1 (**S1 Table) (S1 Fig)** [18]. CTV-1 leukemic cells also present co-stimulatory ligands for NKG2D, DNAM-1, CD2, and LFA-1, but strongly express MHC class I molecules [4]. Co-incubating NK cells with CTV-1 downregulated the expression of all activation receptors aside from CD57 and NKp44, to the extent that there were significant reductions in the proportions of NK cells expressing these receptors (p < 0.04). We further investigated the effect of CTV-1 stimulation on NK cell activation receptor expression and observed an increase in the percentage of cells expressing the activation markers CD25 (mean increase 28.1% ± 9.55; p = 0.003) and CD69 (mean increase 38.6% ± 14.8; p = 0.004), but no significant change in the expression of other activation receptors, relative to unstimulated NK cells was observed **(Fig 1B).**

NK cell stimulation with the insensitive lymphoma cell line Daudi induced a small but significant reduction in the proportion of NK cells expressing 2B4, NKp46, and NKp80 (p < 0.03) but slightly increased the number of NK cells expressing NKp30 relative to NK cells incubated in medium alone (**Fig 1A)**. Daudi cells exhibit low to no MHC Class I expression and express fewer ligands for the engagement of NK cell activation receptors such as NKG2D and DNAM-1 relative to the leukemic cell lines K562 and CTV-1 (**S1 Table)**. Co-incubating NK cells with the MHC Class I positive, NK- sensitive myeloma cell line RPMI-8226 reduced the percentage of CXCR4^+^ NK cells (mean decrease 50.65% ± 20.38; p = 0.005) without significant change in the expression of other activation receptors compared to unstimulated NK cells (**Fig 1A)**. NK cell exposure to the MHC class I positive, NK-insensitive breast adenocarcinoma cell line MCF-7 cells did not affect the expression of NK cell activation receptors relative to NK cells incubated in medium alone. Since the presence of soluble factors such as TGF-β in the tumor microenvironment (TME) can significantly affect NK cell phenotype and function in response to target cell stimulation, we analyzed TGF-β1 content in the supernatant of tumor target cells incubated alone or co-cultured with NK cells but found no detectable levels of TGF-β1 secretion (**S2 Fig)** [19, 20].

Exposure to IL-2, IL-12, IL-15, or IL-12/15/18 induced a similar “trafficking” signature to that induced by tumor cells: CXCR4 and CD62L expression was downregulated compared to unstimulated NK cells. Exposure to IL-2 slightly increased the percentage of NK cells expressing 2B4 compared to unstimulated NK cells (p = 0.03). Although IL-18 did not significantly affect NK cell receptor expression when used alone, exposure to IL-12/15/18 increased the percentage of NK cells expressing NKp30 (mean increase 31.05% ± 14.43; p = 0.009) compared to NK cells incubated in medium alone. Thus, priming with different target tumor cells and cytokines induces distinct and differential NK cell antigen expression profiles.

### Tumor-priming promotes the rapid secretion of pro-inflammatory cytokines by NK cells

**Fig 2:**
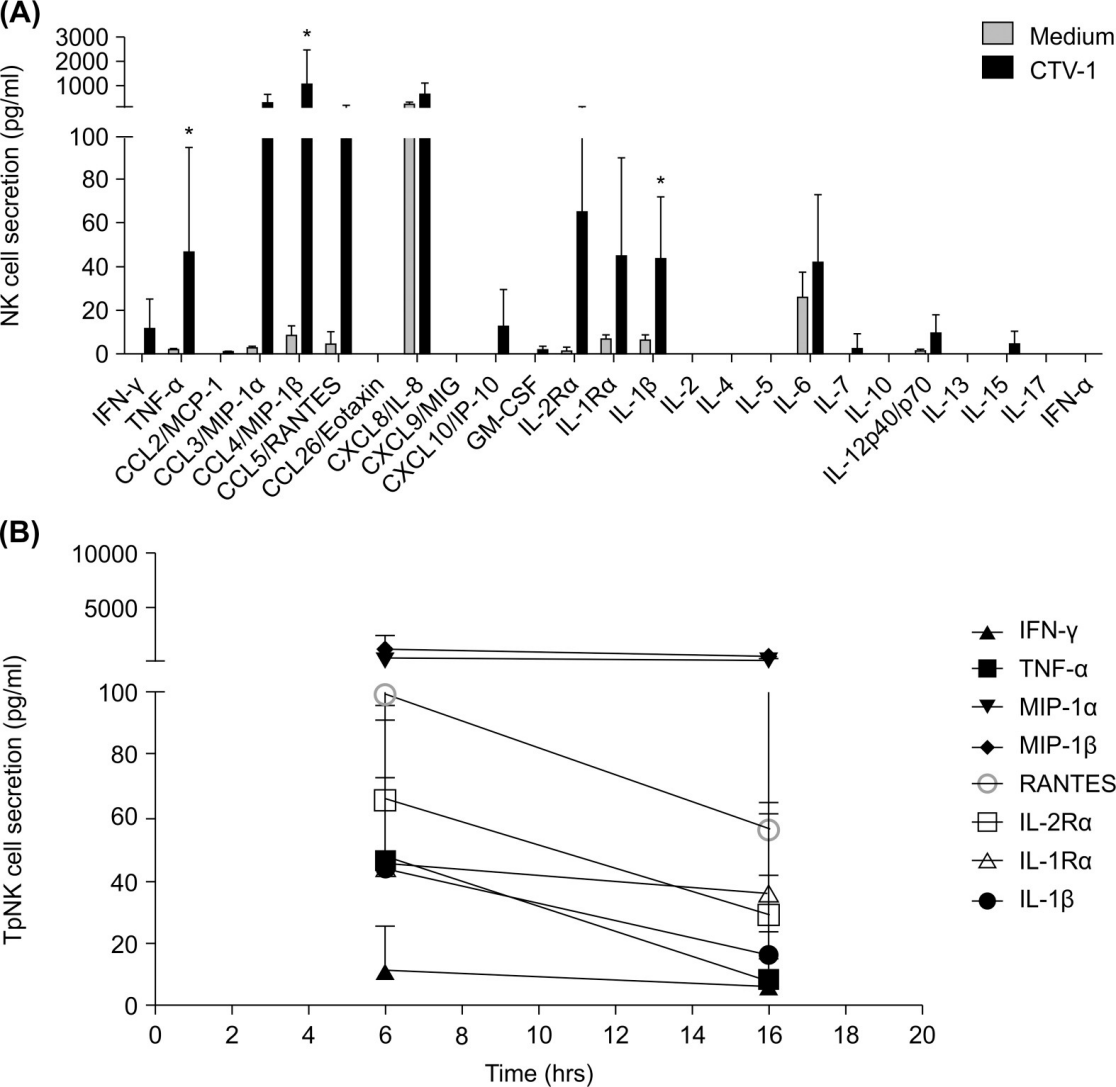
Influence of CTV-1 cell priming on pro-inflammatory cytokine secretion by NK cells. (A) Freshly isolated NK cells were incubated alone or with CTV-1 cells for 6 hours at 37°C. Supernatants were harvested, and the concentrations of 25 different cytokines and chemokines were determined using a multiplex immunoassay. Bars represent means ± SD of 4 different donors. Post-stimulation cytokine secretion by NK cells was compared with that of cells incubated in medium alone using the paired *t-*test. Statistical significance is indicated as: *P <0.05; **P <0.01; ***P <0.001. (B) NK cells were incubated with CTV-1 cells at 37°C. Supernatants were harvested at different time points, as indicated, and the concentrations of IFN-γ, TNF-α, MIP-1α, MIP-1β, and RANTES were determined by a multiplex immunoassay. Values represent mean ± SD of 4 different donors.

We investigated the influence of target cell recognition on the release of soluble mediators by NK cells. Previous studies have defined the cytokine profile of NK cells following target cell stimulation with K562 [10]. Although CTV-1 cells induced an NK cell antigen expression profile that was similar to that induced by K562 cells, we have previously shown that CTV-1 priming enhances the cytolytic function of NK cells *in vitro*, whereas K562-mediated priming has no effect on cells’ cytolytic capacity [4]. We analyzed cytokine release by NK cells following their exposure to CTV-1 cells. Resting NK cells were co-incubated in medium alone or with CTV-1 cells for 6 h. Supernatants were harvested, and the concentration of 25 different soluble factors was determined by a multiplex immunoassay **(Fig 2A)**. Low to moderate levels of the chemokines MIP-1α (CCL3), MIP-1β (CCL4), RANTES (CCL5), and IL-8 (CXCL8) and the cytokines IL-1Rα, IL-1β, and IL-6 were detected in supernatants from NK cells incubated without target cells, with CTV-1 stimulation inducing a 107-, 124-, 22-, 3-, 7-, 7-, and 2-fold increase in their secretion, respectively. CTV-1 stimulation of NK cells also induced the secretion of low to moderate levels of IFN-γ (p = 0.02), TNF-α (p = 0.03), IL-2Rα, IFN-γ-inducible protein (IP)-10 (or CXCL10), and IL-12p40/p70. In contrast to previous studies using cytokine-cultured NK cells [21, 22], incubation with CTV-1 cells had minimal or no effect on the secretion of granulocyte-macrophage colony-stimulating factor (GM-CSF), IL-5, and IL-13. In contrast to previous studies using K562-stimulated NK cells [10], CTV-1 stimulation of NK cells did not induce the secretion of detectable levels of monocyte chemoattractant protein (MCP)-1 (or CCL2), monokine induced by gamma-interferon (MIG) (or CXCL9), IL-7, or IFN-α. IL-2, IL-4, IL-5, IL-13, IL-15, IL-17, and eotaxin (CCL26) secretion by NK cells was not detected. Similar to previous studies on target cell recognition of K562 by NK cells [10], incubating NK cells with CTV-1 cells for 6 hours induced greater cytokine secretion by NK cells (**Fig 2B)**.

**Fig 3:**
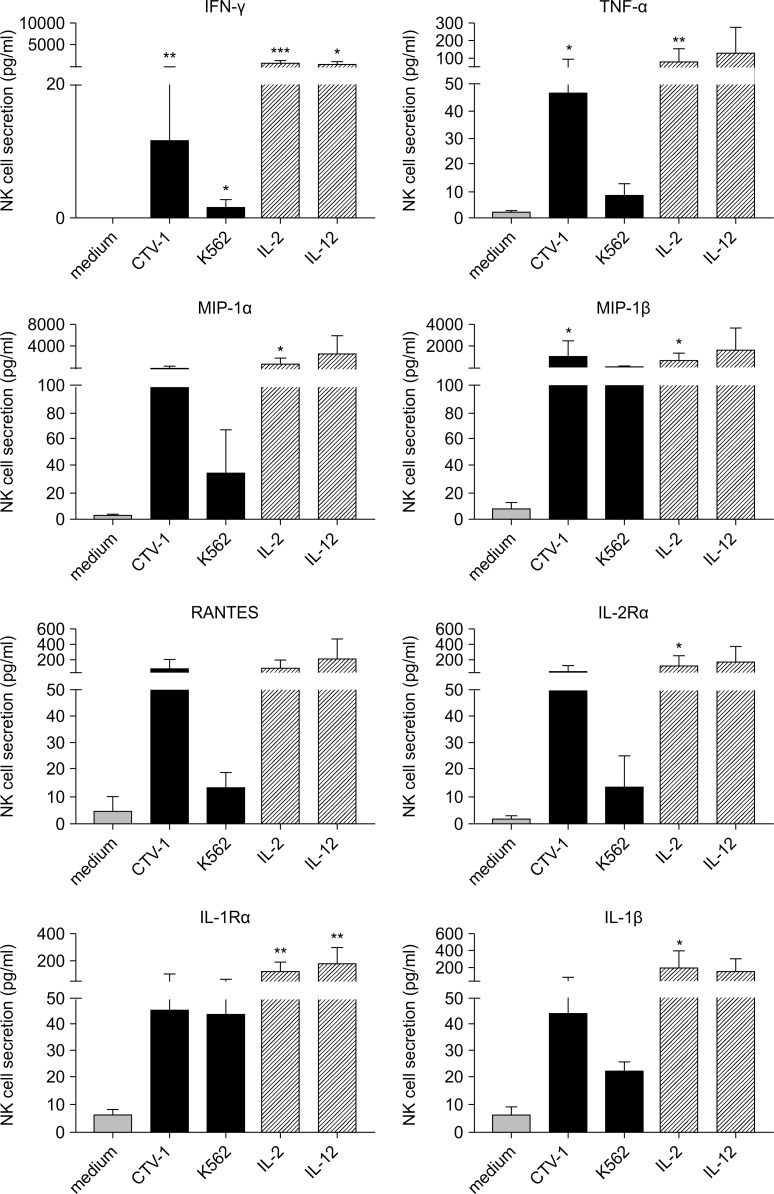
Influence of tumor- or cytokine-priming on cytokine secretion by NK cells. NK cells were incubated in medium alone, with K562, or CTV-1 cells for 6 hours or with IL-2 or IL-12 overnight at 37°C. Supernatants were harvested, and the concentrations of IFN-γ, TNF-α, MIP-1α, MIP-1β, RANTES, IL-R2α, IL-1α, and IL-1β were determined by a multiplex immunoassay. Bars represent means ± SD of 4 different donors. Post-stimulation NK cell cytokine secretion was compared with that in cells incubated in medium alone using the paired *t-*test. Statistical significance is indicated as: *P <0.05; **P <0.01; ***P <0.001.

We next compared the secretion of soluble factors after stimulation of NK cells with different target cells and cytokines. For these studies, resting NK cells were incubated in medium alone, co-incubated with CTV-1 or K562 cells for 6 h or with IL-2 or IL-12 overnight. Supernatants were harvested, and the concentrations of IFN-γ, TNF-α, MIP-1α, MIP-1β, RANTES, IL-R2α, IL-1α, and IL-1β determined by a multiplex immunoassay (**Fig 3**). Levels of soluble factors in the supernatants from CTV-1 and K562 cells that had been incubated alone were subtracted from those that were present in co-cultured NK cells. Interestingly, incubating NK cells with CTV-1 cells induced a consistently greater secretion of IFN-γ, TNF-α, MIP-1α, RANTES, IL-2Rα, and IL-1β than that induced by co-incubation with K562 cells. Cytokine-priming NK cells with IL-2 or IL-12 induced similar or higher levels of cytokine secretion by NK cells compared to co-incubation with CTV-1 cells. Since NK cells can induce the secretion of soluble factors in target cells, we confirmed TpNK cell secretion of cytokines such as IFN-γ, TNF-α, MIP-1α, MIP-1β, and RANTES using flow cytometry, which is consistent with previous studies using CTV-1 and K562 (**S3 Fig)** [4, 10].

### Tumor-priming induces a specific transcriptional signature in NK cells

**Fig 4:**
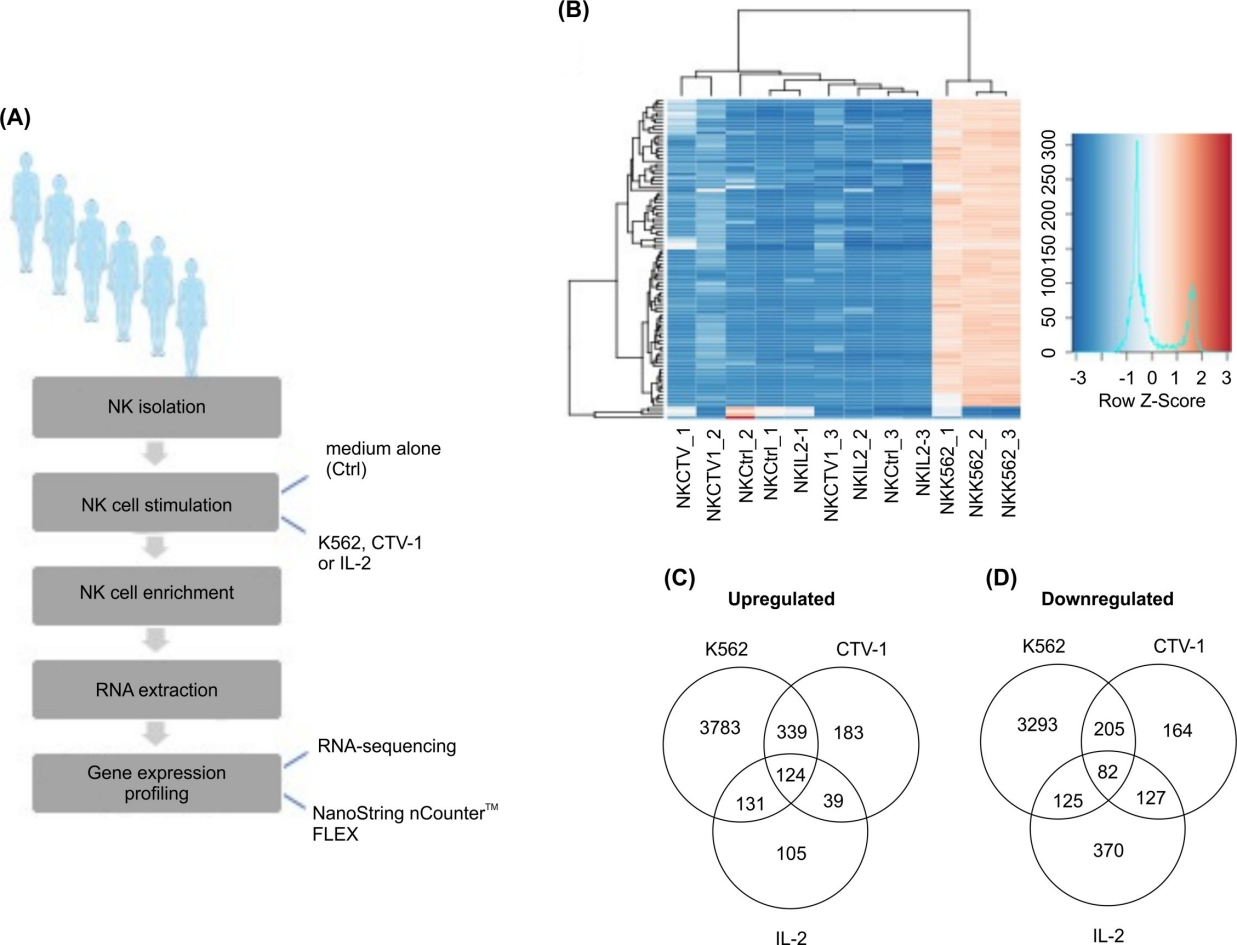
Differential influence of tumor cell or cytokine priming on the transcriptome of NK cells. (A) Schematic of the workflow in obtaining NK cell gene expression profiles after tumor cell or cytokine priming. Human NK cells were isolated from peripheral blood and incubated in medium alone, mitomycin-C-treated K562, or CTV-1 for 6 h or with IL-2 overnight. NK cells were enriched from co-cultures, and RNA extraction and gene expression profiling were performed using RNA-Seq or the NanoString nCounter FLEX platform. (B) Hierarchical clustering was applied to the gene expression values, and the top differentially expressed genes are shown in a heat map with rows representing the relative expression of each gene across 12 samples. Red corresponds to higher gene expression, and blue corresponds to lower gene expression. Similarity in gene expression patterns between different samples are reflected in the resulting dendrogram. Z-Score is calculated using the following formula: (gene expression value in sample of interest)—(mean expression across all samples) / SD. Venn diagrams generated by the intersection of list of genes upregulated (C) or downregulated (D) by at least 1 Log2fold (P <0.05) vs. NK cells incubated in medium alone are shown.

NK cell activation is associated with simultaneous changes in the expression of multiple genes [6]. We studied the influence of exposure to tumor targets or cytokines on the NK cell transcriptome. For this, resting NK cells were incubated in medium alone, with mitomycin C–treated K562 or CTV-1 cells for 6 hours, or with IL-2 overnight, after which RNA was extracted from NK cells that had been isolated from the co-culture. Gene expression was analyzed using RNA-Seq or the NanoString nCounter FLEX amplification-free gene expression profiling platform (**Fig 4A)**. RNA-Seq analysis revealed that stimulation with K562, CTV-1, or IL-2 induced the expression of a number of common genes by NK cells (e.g., *SOCS1/2*, *ULBP1/2*, *SIGLEC12*, *E2F1/2*, *BCL21*, *CDCA5*, *WEE1*, *EGR1*, *HMGB3*), compared to NK cells incubated in medium alone (**S2 Table**). Following stimulation, the most upregulated gene was *HSPA1A*—the intracellular expression of which is linked to the promotion of protein chaperoning, transport, and folding of naïve and mutated proteins and cytoprotection in the presence of stress or activation stimuli (**S3–S5 Tables)** [23]. Functional analysis using available GO annotations in public databases suggests that the observed overlap in gene expression is reflective of common cellular and metabolic processes after target cell or cytokine stimulation (**S4 Fig)**.

A specific tumor-induced gene expression signature was observed in NK cells after exposure to K562 or CTV-1 targets. The gene expression profile of NK cells in which cytotoxicity has been fully triggered through exposure to NK-sensitive K562 cells exhibits the overexpression of many genes relative to resting NK cells or NK cells that have been primed for subsequent killing by co-incubation with the less sensitive target cell line CTV-1 or IL-2 (**Fig 4B)**. Incubation with K562 cells also exclusively induced the downregulation of numerous genes including *GZMA/B*, *HAVCR1/2*, *JAK1/3*, *MAP3K2/3/5/9/10/12/13* relative to unstimulated NK cells, which may explain previous reports on NK cell functional inactivation after K562 stimulation [11–13]. CTV-1 stimulation of NK cells affected many genes common to K562 stimulation including the upregulation of *CCNB1/2*, *VGF*, *STAT5A*, *MARCKSL1*, *ICAM5*, and *FUT3* relative to NK cells incubated alone (**S6 Table)**. The influence of NK-target cell interactions induced the largest overlap in the transcriptome of NK cell across all conditions with 544 common genes exclusively affected by K562 or CTV-1 stimulation but not IL-2 (**Fig 4C and 4D**). The Ingenuity pathway analysis also revealed an overlap in specific biological functions, canonical pathways, and upstream regulators associated with the transcriptional responses of NK cells activated through target cell recognition (**S5 Fig)**.

**Fig 5:**
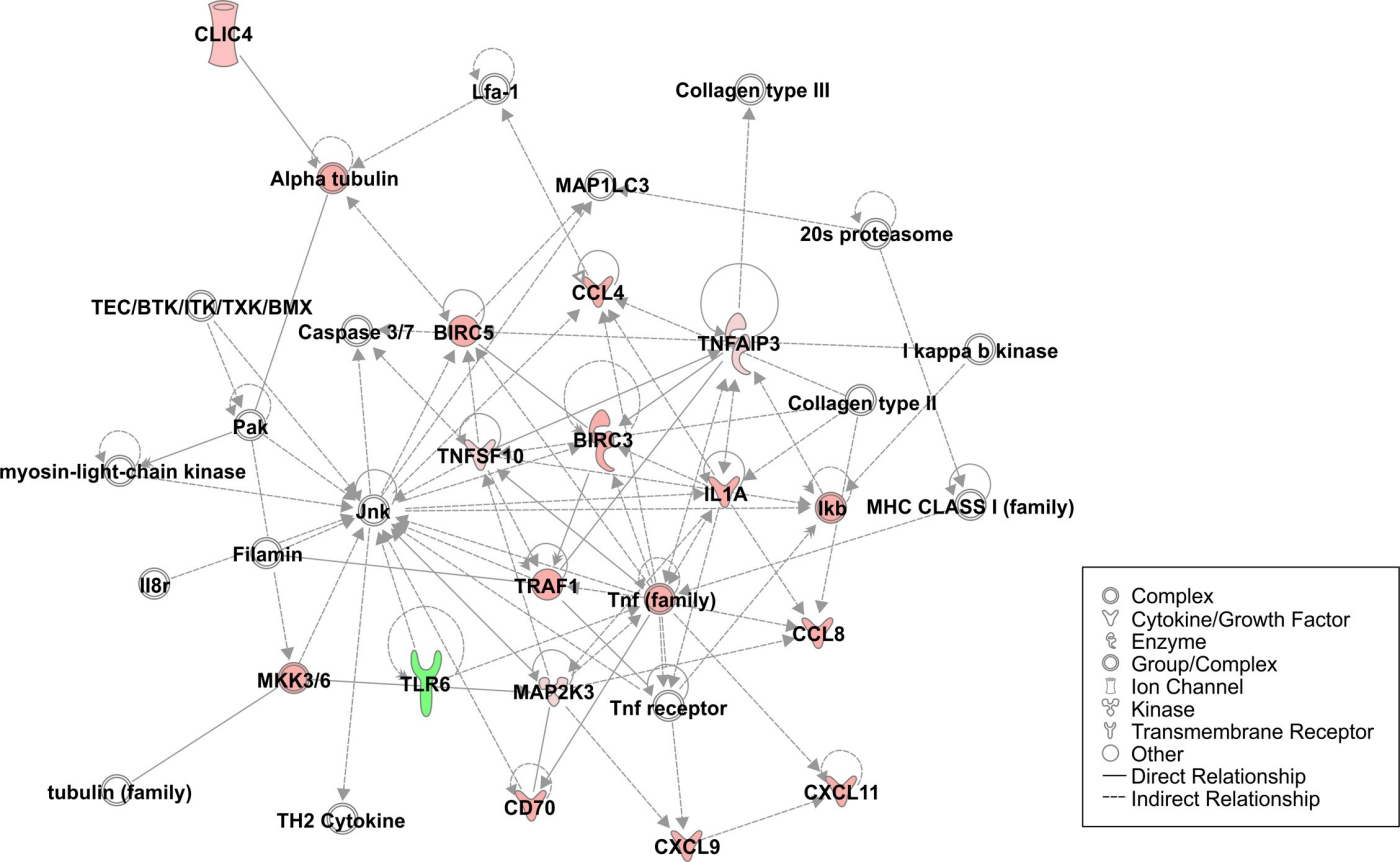
Top cell signaling network induced in tumor-primed NK (TpNK) cells as generated by Ingenuity pathway analysis. Nodes represent genes that are either upregulated (red), downregulated (green), or absent from our original differential expression analysis (white), but play an important role in the tumor-specific signaling network according to Ingenuity Pathway Analysis. The intensity of node color signifies the extent of change in expression of the respective gene relative to NK cells incubated in medium alone. Node shapes and the relationships between different genes are outlined in the figure legend.

To further investigate NK cell transcriptional responses associated with enhanced subsequent killing of tumor targets, NK cell gene expression profiles were analyzed using NanoString nCounter FLEX amplification-free gene expression profiling platform after NK cell incubation in medium alone, with CTV-1 cells, or with IL-2. Consistent with the RNA-Seq results, the gene expression profiles of NK cells after exposure to CTV-1 or IL-2 partially overlapped with the upregulation of genes involved in enhanced effector functions such as cytotoxicity (e.g., *FAS*, *TNSF10*, *CD69*, *CD80*, *CD83*, and *MAPK11*) and cytokine secretion (e.g., *TNF*, *IFNG*, *CCL3/4/8*, and *CXCL9/11*) (**S7 Table**). However, tumor-priming with CTV-1 cells preferentially induced overexpression of genes including *CCNB2*, *DUSP2*, *MAP2K3*, *MARCKSL1*, *STAT5A*, and *TNFAIP3*. **Table 1** lists the genes for which the expression is influenced by incubation with CTV-1 cells or IL-2 and the biological functions associated with their expression. The majority of genes preferentially overexpressed in LAK cells (including *IL-6*, *CCL1/20/22*, *CCR7*, and *CXCL1/2/3/5/8*) are associated with cytokine/chemokine molecules involved in cell-to-cell signaling. These findings align with previous studies reporting on the IL-2 activation of NK cells [24]. In contrast, the top biological functions associated with the expression of genes that are exclusively influenced by tumor-priming relate to cell death and survival. The top signaling network associated with the transcriptome of tumor-primed NK cells is shown in **Fig 5** and demonstrates the central role of genes exclusively affected by CTV-1 stimulation in the tumor-priming pathway. Although gene expression data show differences in NK cell responses to tumor-priming, post-transcriptional mechanisms can highly impact the protein repertoire of cells. Crucial molecules exclusively upregulated by TpNK cells like CD70 and CD137 (TNFRSF9 or 4-1BB) were analyzed using flow cytometry to demonstrate NK cell upregulation of expression following target cell recognition (**S6 Fig)**. However, other signaling molecules such as MAP2K3, STAT5a, TNFAIP3, and VEGF showed no difference in protein expression between resting and primed NK cells. The relationship between mRNA and protein levels can vary depending on the dynamic state of the cells, and other factors like protein half-life and post-translational mechanisms can play a role in the observed differences between mRNA and protein levels [25]. Thus, in many cases, a delay between observed differences in transcriptional induction and protein levels should be considered. Future functional studies of key genes regulating TpNK signaling pathways identified in this paper may identify new biomarkers or reveal previously unknown drivers of NK cell anti-tumor function.

**Table 1:** NK cell gene expression exclusively affected by CTV1- or IL-2-priming.

NK cell stimulus	Genes affected (vs medium)	Top biological functions
CTV-1	Upregulated: *BIRC5*, *CCNB2*, *CD70*, *CXCL10*, *DUSP2*, *DUSP6*, *EEF1A1*, *EGR2*, *EGR3*, *FGFR1*, *FLT1*, *GPR183*, *HIST2H2AA3*, *IL4I1*, *INSIG1*, *MAP2K3*, *MARCKSL1*, *NDC80*, *NR4A2*, *RHOC*, *RHOG*, *SERPINE1*, *STAT5A*, *TNFAIP3*, *TNFSF9*, *TOP2A*, *TTK* and *VEGFA*. Downregulated: *CCR1*, *FCGR2A*, *NCF2*, *RAB3D*, *SERPINB2* and *THB*.	Cell death and survival, cellular function and maintenance, cellular movement, cellular development, cellular growth and proliferation
IL-2	Upregulated: *ACTB*, *ADAMTS1*, *ADORA2A*, *AGK*, *ARF6*, *BATF*, *CALR*, *CASP7*, *CCL1*, *CCL20*, *CCL22*, *CCR7*, *CD274*, *CD38*, *CD40*, *CD40LG*, *CEBPB*, *CEBPG*, *CSF1*, *CXCL1*, *CXCL2*, *CXCL3*, *CXCL5*, *CXCL8*, *CYTIP*, *DPP4*, *ELOVL6*, *FURIN*, *G6PD*, *GCH1*, *GZMA*, *HAVCR1*, *HIF1A*, *ID2*, *IL27RA*, *IL3RA*, *IL6*, *IRF*, *ISG15*, *ITGB7*, *JAK3*, *LAG3*, *LAMP3*, *LDLR*, *LTB*, *MAFF*, *MSC*, *MX1*, *MX2*, *PDE4A*, *PSMB9*, *PSME2*, *PTGER2*, *RIPK2*, *SERPINB2*, *STAT1*, *STAT3*, *TAP1*, *TAP2*, *TAPBP*, *TNFRSF1B*, *TNFSF4*, *TRAF2*, *TRAFD1*, *TREX1* and *VASP*. Downregulated: *AIF1*, *AOAH*, *C3AR1*, *CD27*, *CD84*, *CEBPA*, *CLEC7A*, *CXCR2*, *CXCR4*, *FCGR2B*, *HDAC5*, *HLA-DMA*, *JAML*, *LAPTM5*, *MRC1*, *OLR1*, *PTGDR*, *RASAL1*, *S1PR1*, *SMARCD3*, *SPTBN1*, *TLR1* and *TYROBP*.	Cell-to-cell signaling and interaction, cellular movement, cellular function and maintenance, cellular growth and proliferation, cellular development

## Discussion

NK cell recognition of tumor targets can involve a wide array of cell surface receptors, which trigger downstream signal transduction pathways that are ultimately integrated to determine activity [2]. The ability of NK cells to mediate cytotoxicity against tumor cells co-evolved alongside tumor escape mechanisms that facilitate tumorigenesis without triggering the signaling threshold for full NK cell activation. Tumor resistance can be overcome by priming NK cells *in vitro* via two mechanisms: cytokine stimulation and target cell recognition [26]. In this study, we report loss of activation receptor expression, rapid pro-inflammatory cytokine secretion, and tumor-specific gene expression signature induction in NK cells following priming with tumor targets, which is independent of cytokine stimulation.

NK cells isolated from patients with cancer commonly display low levels of activation receptors, which has been associated with NK cell dysfunction [15, 27–30]. Exposing NK cells to targets has been shown to downregulate the expression of several activation receptors, including NKG2D, CD16, NKp30 and NKp46. NKG2D downregulation occurs as a consequence of its engagement by membrane-bound or soluble ligands to induce clatherin-mediated endocytosis and activation of the ERK1/2 signaling pathway via the DAP10 signaling adaptor [31, 32]. Exposure to TGF-β is also one of the main mechanisms through which surface expression of NKG2D and NKp30 is downregulated [33, 34]. Other receptors, such as CD16, are downregulated via proteolytic cleavage by A disintegrin and metalloproteinase (ADAM)-17 or matrix metalloproteinase (MMP)-25; we have previously hypothesized that this mechanism enables dimerized CD3-ζ to associate with CD2 in order to provide essential co-stimulation, as first reported by Warren et al. over 20 years go [4, 17, 35, 36]. Although the downregulation of activation receptors has been proposed as a regulatory mechanism to dampen NK cell activity, our group and others have previously reported that this NK cell phenotype is associated with enhanced NK cell cytotoxicity against tumor targets [3, 4, 17, 37–39]. We have also shown that tumor-priming NK cells induces the expression of NK cell activation markers such as CD69 and CD25; the secretion of pro-inflammatory cytokines, including MIP-1 α /β, RANTES, IL-6, IL-8, and IL-1β; and upregulation of numerous genes that are associated with enhanced NK cell cytotoxicity and immunomodulatory functions (including *FAS*, *TNFSF10*, *CD80*, *MAPK11*, *TNF*, *IFNG*, *CCL3/4/8*, and *CXCL9/11*).

It is possible that NK cells involved in tumor recognition downregulate surface receptors as part of the priming process and that the internalization or cleavage of these from the cell surface is essential for optimal NK cell function. Through the use of pharmacological inhibitors and NK cells transfected to express a non-cleavable form of CD16, receptor shedding was recently shown to positively regulate NK cell activity by enhancing survival, motility, and serial engagement of target cells [9]. Although the downregulation of CD16 expression renders NK cells less sensitive to subsequent activation via that receptor, they remain responsive to further stimulation via other receptors. This can explain the responsiveness of NK cells previously primed with CTV-1 to subsequent stimulation with other target cells. Other metalloproteinases, such as ADAM9, were upregulated after tumor recognition and may play a similar role in downregulating the expression of other NK cell receptors, such as NKG2D or NKp46 and promoting NK cell survival and serial engagement with targets [40]. These findings may explain why therapeutic interventions to prevent receptor downregulation through ADAM17 inhibitors have not proved successful thus far. Downregulation of NK cell surface receptors like the bone marrow trafficking receptor CXCR4 after cytokine-priming, can also be strengthened or reversed by other soluble factors in the microenvironment [19]. This might explain why IL-18 had a synergistic effect on CXCR4 downregulation when used with IL-12 and IL-15, relative to NK cell exposure to any of the cytokines alone.

Administering CTV-1-primed NK cells to patients with acute myeloid leukemia has been shown to be more clinically effective than IL-2 activated NK cells [41, 42]. Insight into the mechanism(s) underlying these different clinical responses is provided by the differential transcriptional activity in NK cells, which is triggered by co-incubation with CTV-1 cells or IL-2 stimulation. Consistent with previous studies, the majority of genes that are preferentially overexpressed after exposure to IL-2 are involved in cell-to-cell signaling, whereas CTV-1 stimulation upregulated the expression of genes that are specifically associated with NK cell cytotoxicity against tumor targets (including *STAT5A*, *MAP2K3*, *DUSP2/6)* [24]. For example, the JAK-STAT signaling pathway, which is activated by both classes of stimuli, triggers STAT5A in TpNK cells, which plays a specific role in regulating immune responses to hypoxic stress in the TME via the prolyl-4-hydroxylase domain enzyme (PHD)-3 and hypoxia-inducible factors (HIF) [43]. Other stress signals associated with cancer progression lead to the activation of the MAPK pathway, which plays a critical role in NK cell lysis of tumor cells by mobilizing granule components upon contact with NK cell-sensitive target cells [44]. This crucial pathway is tightly regulated by several factors, including DUSP2/6 [45]. Pal et al. recently reported enhanced tumor-specific NK cell cytotoxicity after tumor-priming with leukemic patient specimens, which is linked to differences in gene expression compared to cytokine-priming as well as the induction of memory-like function [46]. We identified other genes in the transcriptome of tumor-primed NK cells that are likely to play a role in NK cell anti-tumor responses that should be further investigated.

Previous studies characterizing tumor-associated NK cell responses using NK cells isolated from patients with cancer and expanded *ex vivo* using IL-2 and an irradiated EBV-transformed lymphoblastoid cell line demonstrated the overexpression of genes such as *CCNB2*, *CCL4*, *MIF*, *and TUBB*, which were shown here to be upregulated after exposure to tumor targets [47]. Moreover, these overexpressed genes were exclusively activated by IL-2 (e.g., *CXCL5*, *GZMA*, and *LTB*). It is possible that NK cells activated by simultaneous or sequential exposure to cytokines and target cells exhibit gene expression signatures that are an amalgam of the profiles observed in this study. Thus, our *in vitro* observations of changes in gene or receptor expression in response to the independent effects of different tumor cells or cytokines may not be recapitulated in the TME *in vivo* in which both classes of stimuli are present. Nonetheless, characterizing the NK cell activation phenotype after exposure to tumor targets, which is distinct to and independent of cytokine stimulation, is an important step towards delineating the specificity of NK cell anti-tumor responses.

NK cells are fast-acting lymphocytes, which is partially explained by high level of transcriptional activity in their resting state [48]. Upon NK cell stimulation, protein levels can drastically increase without substantial changes to their respective mRNA levels. Thus, it is possible that priming resting NK cells enhances NK cell function by promoting the translation of readily abundant mRNA transcripts. In this study, the transcriptional activity for some genes that are central to anti-tumor effector responses, such as *GZMA*, was not influenced after target cell stimulation. A possible explanation may be that these genes are constitutively highly expressed in resting NK cells and that target stimulation increases translation rather than transcription.

Differential NK cell phenotypic and transcriptional signatures that are induced after exposure to tumor cells or cytokines highlight the complex nature of NK cell signaling and the importance of both the target cell and cytokine milieu. Study limitations dictate that, until our findings are replicated with more donors using different tumor targets and cytokine ratios and concentrations indicative of the *in vivo* setting, speculation about *in vivo* implications of NK cell responses remains premature. Previous reports of impaired NK cell function in patients with cancer may be partly influenced by chronic exposure of peripheral blood NK cells to tumor cells, thereby leading to a downregulation of activating ligands. Maintenance of patient-derived NK cells in IL-2, which is a common laboratory practice, is likely to influence the phenotype and transcriptome of NK cells and explain the disparities in the published literature. Future studies should aim to further contribute to our understanding of tumor-specific NK cell responses by characterizing the expression of inhibitory receptors involved in immune checkpoints and the priming responses of different NK cell subpopulations involved in tumor recognition. Increasing evidence for a specific tumor-induced signature in NK cell responses may have important implications in the clinic and key signaling molecules identified to play a specific role in tumor target recognition by NK cells can be targeted to deliver effective therapeutic approaches for the treatment of cancer.

## Supporting information

S1 Fig Expression of NK cell ligands on different tumor cell lines. Tumor cell lines K562, CTV-1, Daudi, RPMI-8226 and MCF-7 incubated alone at 37°C in 5% v/v CO were assessed for their expression of different NK cell ligands using flow cytometry. The experiment was performed three times and representative plots are shown with light grey histograms representing FMO controls and dark grey histograms representing stained samples.
(TIF)

S2 Fig Analysis of human TGF-β1 secretion by tumor cells and NK cells. Tumor cell lines K562, CTV-1, Daudi, RPMI-8226, and MCF-7 were incubated alone or with freshly-isolated, resting NK cells for 16 hours at 37°C in 5% v/v CO_2_. Thereafter, cell-free supernatants were collected and stored at −80°C pending analysis using a Quantikine ELISA kit according to manufacturer’s instructions. Optical density values from standard controls were used to interpolate X values of secretion measurements in pg/ml following baseline correction using blank controls. Tumor cell lines cultured alone or with NK cells did not secrete detectable levels of TGF-β1.
(TIF)

S3 Fig Intracellular staining of NK cell cytokine secretion following tumor-priming. Freshly isolated NK cells were incubated in medium alone or with CTV-1 cells for 6 hours at 37°C. Then, cells were surface-stained with fluorochrome-conjugated anti-CD56 and anti-CD3 mAbs. Cells were fixed, permeabilized, and intracellularly stained with fluorochrome-conjugated mAbs against IFN-γ, TNF-α, MIP-1α, MIP-1β, and RANTES. Brefeldin A and/or monensin were added when appropriate 1 hour after incubation. Appropriate isotype and FMO controls were included in each experiment. (A) Bars represent mean percentage NK cell expression ± SD of 3–7 different donors. (B) Bars represent mean NK cell median fluorescence intensity values ± SD of 3 different donors. NK cell expression after stimulation was compared with cells treated with medium alone using the paired *t-*test. Statistical significance is indicated as: *P <0.05; **P <0.01; ***P <0.001.
(TIF)

S4 Fig Functional analysis of NK cell gene expression overlap after stimulation with K562, CTV-1 or IL-2 using the Panther classification system. Freshly isolated NK cells were incubated in medium alone, with mitomycin-C- treated K562 or CTV-1 cells for 6 hours, or with IL-2 overnight. Then, NK cells were selectively enriched from the co-culture, mRNA was extracted, and gene expression was analyzed using RNA-sequencing. Functional annotation analysis using gene ontology terms was performed on genes commonly upregulated after exposure to K562, CTV-1, or IL-2 relative to NK cells incubated in medium alone that met the cutoff values (>1 Log2fold change, P-value <0.05) using the Panther Classification System gene list analysis tool.
(TIF)

S5 Fig Ingenuity pathway analysis heatmaps of top biological functions, canonical pathways, and upstream regulators associated with NK cell gene expression profiles after stimulation with K562, CTV-1, or IL-2. Freshly isolated NK cells were incubated in medium alone, with mitomycin-C-treated K562 or CTV-1 cells for 6 hours, or with IL-2 overnight. Then, NK cells were selectively enriched from the co-culture, mRNA was extracted, and gene expression was analyzed using RNA-sequencing. Biological functions, canonical pathways, and upstream regulators associated with NK cell genes variably expressed after exposure to K562, CTV-1, or IL-2 relative to NK cells incubated in medium alone that met the cutoff values (>1 Log2fold change, P-value <0.05) were analyzed using Ingenuity pathway analysis software. Colors indicate predicted activity according to activation z-scores, with orange predicting an overall increase and blue indicating a decrease in activity relative to NK cells incubated in medium alone.
(TIF)

S6 Fig NK cell expression of tumor-associated signaling molecules following co-incubation with K562, CTV-1, or IL-2. Freshly isolated NK cells were incubated in medium alone or with K562 or CTV-1 cells for 6 hours or IL-2 overnight at 37°C. Then, cells were surface stained with fluorochrome-conjugated anti-CD56, anti-CD3, anti-CD137, and anti-CD70 mAbs. Cells were fixed, permeabilized, and intracellularly stained with fluorochrome-conjugated mAbs against STAT5a, MAP2K3, TNFAIP3, and VEGF. Brefeldin A and/or monensin were added when appropriate 5 hours before the end of the incubation period. Appropriate isotype and FMO controls were included in each experiment. (A) Bars represent mean percentage NK cell expression ± SD of 3–5 different donors. (B) Bars represent mean NK cell median fluorescence intensity values ± SD of 3–5 different donors.
(TIF)

S1 Table Expression of ligands for NK cell receptors on different tumor cell lines. (DOCX)

S2 Table Overlap in NK cell gene expression from RNA-sequencing analysis after stimulation with K562, CTV-1, or IL-2. (DOCX)

S3 Table Top 50 variably expressed NK cells genes according to log2fold change from RNA-sequencing analysis after NK cell exposure to K562 cells. (DOCX)

S4 Table Top 50 variably expressed NK cells genes according to log2fold change from RNA-sequencing analysis after NK cell exposure to CTV-1 cells. (DOCX)

S5 Table Top 50 variably expressed NK cells genes according to log2Fold change from RNA-sequencing analysis after NK cell exposure to IL-2. (DOCX)

S6 Table Tumour-induced changes from RNA-sequencing analysis in NK cell gene expression after NK cell exposure to K562 or CTV-1. (DOCX)

S7 Table Overlap in NK cell gene expression from NanoString nCounter gene expression profiling after stimulation with CTV-1 or IL-2. (DOCX)

## References

[1] Bryceson YT, March ME, Ljunggren HG, Long EO. Synergy among receptors on resting NK cells for the activation of natural cytotoxicity and cytokine secretion. Blood. 2006;107(1):159–66. 2005-04-1351 [pii]; 10.1182/blood-2005-04-1351 16150947 PMC1895346

[2] Morvan MG, Lanier LL. NK cells and cancer: you can teach innate cells new tricks. Nat Rev Cancer. 2016;16(1):7–19. 10.1038/nrc.2015.5 . 26694935

[3] North J, Bakhsh I, Marden C, Pittman H, Addison E, Navarrete C, et al Tumor-primed human natural killer cells lyse NK-resistant tumor targets: evidence of a two-stage process in resting NK cell activation. JImmunol. 2007;178(1):85–94. 178/1/85 [pii]. 17182543 10.4049/jimmunol.178.1.85

[4] Sabry M, Tsirogianni M, Bakhsh IA, North J, Sivakumaran J, Giannopoulos K, et al Leukemic priming of resting NK cells is killer Ig-like receptor independent but requires CD15-mediated CD2 ligation and natural cytotoxicity receptors. JImmunol. 2011;187(12):6227–34. jimmunol.1101640 [pii]; 10.4049/jimmunol.1101640 22084431

[5] Meazza R, Azzarone B, Orengo AM, Ferrini S. Role of common-gamma chain cytokines in NK cell development and function: perspectives for immunotherapy. J Biomed Biotechnol. 2011;2011:861920 10.1155/2011/861920 21716670 PMC3118299

[6] Wang F, Tian Z, Wei H. Genomic expression profiling of NK cells in health and disease. Eur J Immunol. 2015;45(3):661–78. Epub 2014/12/06. 10.1002/eji.201444998 . 25476835

[7] Bottcher A, Ostwald J, Koczan D, Knecht R, Kramp B, Dommerich S. Gene expression profiling of circulating natural killer cells in head and neck squamous cell carcinoma. Cancer Genomics Proteomics. 2013;10(5):197–207. . 24136972

[8] Gillard-Bocquet M, Caer C, Cagnard N, Crozet L, Perez M, Fridman WH, et al Lung tumor microenvironment induces specific gene expression signature in intratumoral NK cells. Front Immunol. 2013;4:19 10.3389/fimmu.2013.00019 23382731 PMC3563113

[9] Srpan K, Ambrose A, Karampatzakis A, Saeed M, Cartwright ANR, Guldevall K, et al Shedding of CD16 disassembles the NK cell immune synapse and boosts serial engagement of target cells. J Cell Biol. 2018;217(9):3267–83. Epub 2018/07/04. 10.1083/jcb.201712085 29967280 PMC6122987

[10] Fauriat C, Long EO, Ljunggren HG, Bryceson YT. Regulation of human NK-cell cytokine and chemokine production by target cell recognition. Blood. 2010;115(11):2167–76. blood-2009-08-238469 [pii]; 10.1182/blood-2009-08-238469 19965656 PMC2844017

[11] Jewett A, Bonavida B. Target-induced anergy of natural killer cytotoxic function is restricted to the NK-target conjugate subset. Cell Immunol. 1995;160(1):91–7. Epub 1995/01/01. . 7842490 10.1016/0008-8749(95)80013-9

[12] Cavalcanti M, Jewett A, Bonavida B. Irreversible cancer cell-induced functional anergy and apoptosis in resting and activated NK cells. Int J Oncol. 1999;14(2):361–6. Epub 1999/01/26. 10.3892/ijo.14.2.361 . 9917514

[13] Jewett A, Bonavida B. Target-induced inactivation and cell death by apoptosis in a subset of human NK cells. J Immunol. 1996;156(3):907–15. Epub 1996/02/01. . 8558016

[14] Nieto-Velazquez NG, Torres-Ramos YD, Munoz-Sanchez JL, Espinosa-Godoy L, Gomez-Cortes S, Moreno J, et al Altered Expression of Natural Cytotoxicity Receptors and NKG2D on Peripheral Blood NK Cell Subsets in Breast Cancer Patients. Transl Oncol. 2016;9(5):384–91. 10.1016/j.tranon.2016.07.003 27641642 PMC5024335

[15] Kono K, Ressing ME, Brandt RM, Melief CJ, Potkul RK, Andersson B, et al Decreased expression of signal-transducing zeta chain in peripheral T cells and natural killer cells in patients with cervical cancer. ClinCancer Res. 1996;2(11):1825–8. 9816136

[16] Fauriat C, Mallet F, Olive D, Costello RT. Impaired activating receptor expression pattern in natural killer cells from patients with multiple myeloma. Leukemia. 2006;20(4):732–3. 10.1038/sj.leu.2404096 . 16437151

[17] Romee R, Foley B, Lenvik T, Wang Y, Zhang B, Ankarlo D, et al NK cell CD16 surface expression and function is regulated by a disintegrin and metalloprotease-17 (ADAM17). Blood. 2013;121(18):3599–608. blood-2012-04-425397 [pii]; 10.1182/blood-2012-04-425397 23487023 PMC3643761

[18] Warren HS, Altin JG, Waldron JC, Kinnear BF, Parish CR. A carbohydrate structure associated with CD15 (Lewis x) on myeloid cells is a novel ligand for human CD2. JImmunol. 1996;156(8):2866–73. 8609406

[19] Casu B, Dondero A, Regis S, Caliendo F, Petretto A, Bartolucci M, et al Novel Immunoregulatory Functions of IL-18, an Accomplice of TGF-beta1. Cancers (Basel). 2019;11(1). Epub 2019/01/16. 10.3390/cancers11010075 30641867 PMC6356463

[20] Foltz JA, Moseman JE, Thakkar A, Chakravarti N, Lee DA. TGFbeta Imprinting During Activation Promotes Natural Killer Cell Cytokine Hypersecretion. Cancers (Basel). 2018;10(11). Epub 2018/11/08. 10.3390/cancers10110423 30400618 PMC6267005

[21] Cuturi MC, Anegon I, Sherman F, Loudon R, Clark SC, Perussia B, et al Production of hematopoietic colony-stimulating factors by human natural killer cells. JExpMed. 1989;169(2):569–83. 10.1084/jem.169.2.569 PMC2189209 2521357

[22] Warren HS, Kinnear BF, Phillips JH, Lanier LL. Production of IL-5 by human NK cells and regulation of IL-5 secretion by IL-4, IL-10, and IL-12. JImmunol. 1995;154(10):5144–52. 7730620

[23] Pockley AG, Henderson B. Extracellular cell stress (heat shock) proteins-immune responses and disease: an overview. Philos Trans R Soc Lond B Biol Sci. 2018;373(1738). Epub 2017/12/06. 10.1098/rstb.2016.0522 29203707 PMC5717522

[24] Hodge DL, Schill WB, Wang JM, Blanca I, Reynolds DA, Ortaldo JR, et al IL-2 and IL-12 alter NK cell responsiveness to IFN-gamma-inducible protein 10 by down-regulating CXCR3 expression. J Immunol. 2002;168(12):6090–8. 10.4049/jimmunol.168.12.6090 . 12055219

[25] Liu Y, Beyer A, Aebersold R. On the Dependency of Cellular Protein Levels on mRNA Abundance. Cell. 2016;165(3):535–50. Epub 2016/04/23. 10.1016/j.cell.2016.03.014 . 27104977

[26] Yokoyama WM, Kim S, French AR. The dynamic life of natural killer cells. Annu Rev Immunol. 2004;22:405–29. 10.1146/annurev.immunol.22.012703.104711 . 15032583

[27] Konjevic G, Mirjacic MK, Vuletic A, Jovic V, Jurisic V, Babovic N, et al Low expression of CD161 and NKG2D activating NK receptor is associated with impaired NK cell cytotoxicity in metastatic melanoma patients. ClinExpMetastasis. 2007;24(1):1–11. 10.1007/s10585-006-9043-9 17295095

[28] Healy CG, Simons JW, Carducci MA, DeWeese TL, Bartkowski M, Tong KP, et al Impaired expression and function of signal-transducing zeta chains in peripheral T cells and natural killer cells in patients with prostate cancer. Cytometry. 1998;32(2):109–19. 10.1002/(SICI)1097-0320(19980601)32:2<109::AID-CYTO6>3.0.CO;2-G [pii]. 9627224

[29] Costello RT, Sivori S, Marcenaro E, Lafage-Pochitaloff M, Mozziconacci MJ, Reviron D, et al Defective expression and function of natural killer cell-triggering receptors in patients with acute myeloid leukemia. Blood. 2002;99(10):3661–7. 10.1182/blood.v99.10.3661 11986221

[30] El-Sherbiny YM, Meade JL, Holmes TD, McGonagle D, Mackie SL, Morgan AW, et al The requirement for DNAM-1, NKG2D, and NKp46 in the natural killer cell-mediated killing of myeloma cells. Cancer Res. 2007;67(18):8444–9. 67/18/8444 [pii]; 10.1158/0008-5472.CAN-06-4230 17875681

[31] Ogasawara K, Hamerman JA, Hsin H, Chikuma S, Bour-Jordan H, Chen T, et al Impairment of NK cell function by NKG2D modulation in NOD mice. Immunity. 2003;18(1):41–51. Epub 2003/01/18. . 12530974 10.1016/s1074-7613(02)00505-8

[32] Quatrini L, Molfetta R, Zitti B, Peruzzi G, Fionda C, Capuano C, et al Ubiquitin-dependent endocytosis of NKG2D-DAP10 receptor complexes activates signaling and functions in human NK cells. Sci Signal. 2015;8(400):ra108 Epub 2015/10/29. 10.1126/scisignal.aab2724 . 26508790

[33] Castriconi R, Cantoni C, Della Chiesa M, Vitale M, Marcenaro E, Conte R, et al Transforming growth factor beta 1 inhibits expression of NKp30 and NKG2D receptors: consequences for the NK-mediated killing of dendritic cells. Proc Natl Acad Sci U S A. 2003;100(7):4120–5. Epub 2003/03/21. 10.1073/pnas.0730640100 12646700 PMC153058

[34] Lee JC, Lee KM, Kim DW, Heo DS. Elevated TGF-beta1 secretion and down-modulation of NKG2D underlies impaired NK cytotoxicity in cancer patients. J Immunol. 2004;172(12):7335–40. Epub 2004/06/10. 10.4049/jimmunol.172.12.7335 . 15187109

[35] Warren HS, Altin JG, Waldron JC, Kinnear BF, Parish CR. A carbohydrate structure associated with CD15 (Lewis x) on myeloid cells is a novel ligand for human CD2. J Immunol. 1996;156(8):2866–73. . 8609406

[36] Peruzzi G, Femnou L, Gil-Krzewska A, Borrego F, Weck J, Krzewski K, et al Membrane-type 6 matrix metalloproteinase regulates the activation-induced downmodulation of CD16 in human primary NK cells. J Immunol. 2013;191(4):1883–94. Epub 2013/07/16. 10.4049/jimmunol.1300313 23851692 PMC3745217

[37] Gati A, Da RS, Guerra N, Escudier B, Moretta A, Chouaib S, et al Analysis of the natural killer mediated immune response in metastatic renal cell carcinoma patients. IntJCancer. 2004;109(3):393–401. 10.1002/ijc.11730 14961578

[38] Penack O, Gentilini C, Fischer L, Asemissen AM, Scheibenbogen C, Thiel E, et al CD56dimCD16neg cells are responsible for natural cytotoxicity against tumor targets. Leukemia. 2005;19(5):835–40. 2403704 [pii]; 10.1038/sj.leu.2403704 15744340

[39] Grzywacz B, Kataria N, Verneris MR. CD56(dim)CD16(+) NK cells downregulate CD16 following target cell induced activation of matrix metalloproteinases. Leukemia. 2007;21(2):356–9; author reply 9. 10.1038/sj.leu.2404499 . 17251901

[40] Peduto L. ADAM9 as a potential target molecule in cancer. Curr Pharm Des. 2009;15(20):2282–7. Epub 2009/07/16. . 19601830 10.2174/138161209788682415

[41] Fehniger TA, Miller JS, Stuart RK, Cooley S, Salhotra A, Curtsinger J, et al A Phase 1 Trial of CNDO-109-Activated Natural Killer Cells in Patients with High-Risk Acute Myeloid Leukemia. Biol Blood Marrow Transplant. 2018;24(8):1581–9. Epub 2018/03/30. 10.1016/j.bbmt.2018.03.019 . 29597002 PMC6232080

[42] Kottaridis PD, North J, Tsirogianni M, Marden C, Samuel ER, Jide-Banwo S, et al Two-Stage Priming of Allogeneic Natural Killer Cells for the Treatment of Patients with Acute Myeloid Leukemia: A Phase I Trial. PLoS One. 2015;10(6):e0123416 Epub 2015/06/11. 10.1371/journal.pone.0123416 26062124 PMC4465629

[43] Casetti L, Martin-Lanneree S, Najjar I, Plo I, Auge S, Roy L, et al Differential contributions of STAT5A and STAT5B to stress protection and tyrosine kinase inhibitor resistance of chronic myeloid leukemia stem/progenitor cells. Cancer Res. 2013;73(7):2052–8. Epub 2013/02/13. 10.1158/0008-5472.CAN-12-3955 . 23400594

[44] Wei S, Gamero AM, Liu JH, Daulton AA, Valkov NI, Trapani JA, et al Control of lytic function by mitogen-activated protein kinase/extracellular regulatory kinase 2 (ERK2) in a human natural killer cell line: identification of perforin and granzyme B mobilization by functional ERK2. J Exp Med. 1998;187(11):1753–65. Epub 1998/06/10. 10.1084/jem.187.11.1753 9607917 PMC2212310

[45] Jeffrey KL, Brummer T, Rolph MS, Liu SM, Callejas NA, Grumont RJ, et al Positive regulation of immune cell function and inflammatory responses by phosphatase PAC-1. Nat Immunol. 2006;7(3):274–83. 10.1038/ni1310 . 16474395

[46] Pal M, Schwab L, Yermakova A, Mace EM, Claus R, Krahl AC, et al Tumor-priming converts NK cells to memory-like NK cells. Oncoimmunology. 2017;6(6):e1317411 Epub 2017/07/07. 10.1080/2162402X.2017.1317411 28680749 PMC5486172

[47] Park KU, Jin P, Sabatino M, Feng J, Civini S, Khuu H, et al Gene expression analysis of ex vivo expanded and freshly isolated NK cells from cancer patients. J Immunother. 2010;33(9):945–55. 10.1097/CJI.0b013e3181f71b81 20948442 PMC3096009

[48] Fehniger TA, Cai SF, Cao X, Bredemeyer AJ, Presti RM, French AR, et al Acquisition of murine NK cell cytotoxicity requires the translation of a pre-existing pool of granzyme B and perforin mRNAs. Immunity. 2007;26(6):798–811. 10.1016/j.immuni.2007.04.010 . 17540585

