# Supplementary figures and images for: Tumor- and cytokine-primed human natural killer cells exhibit distinct phenotypic and transcriptional signatures
Source: PLoS One. 2019 Jun 26;14(6):e0218674. doi: 10.1371/journal.pone.0218674 (PMC6594622; doi:10.1371/journal.pone.0218674)

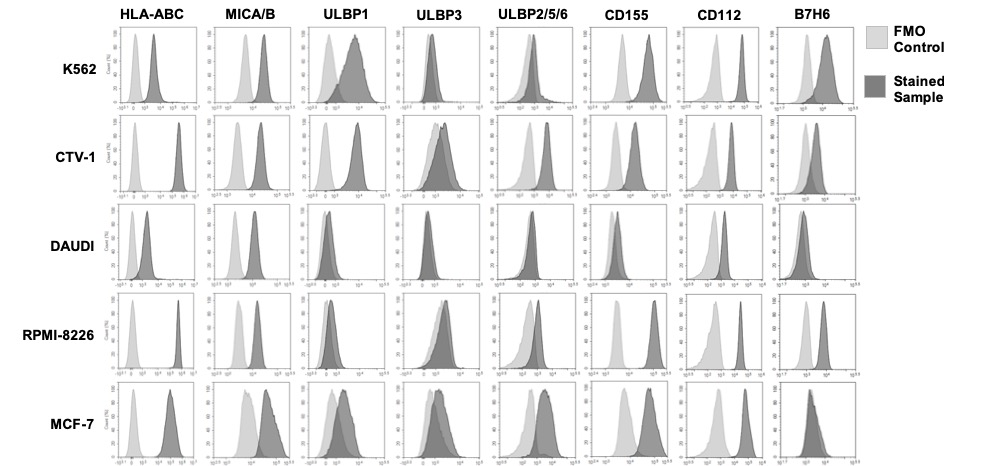

Supplement: S1 Fig — Tumor cell lines K562, CTV-1, Daudi, RPMI-8226 and MCF-7 incubated alone at 37°C in 5% v/v CO were assessed for their expression of different NK cell ligands using flow cytometry. The experiment was performed three times and representative plots are shown with light grey histograms representing FMO controls and dark grey histograms representing stained samples. (TIF) [file pone.0218674.s001.tif]

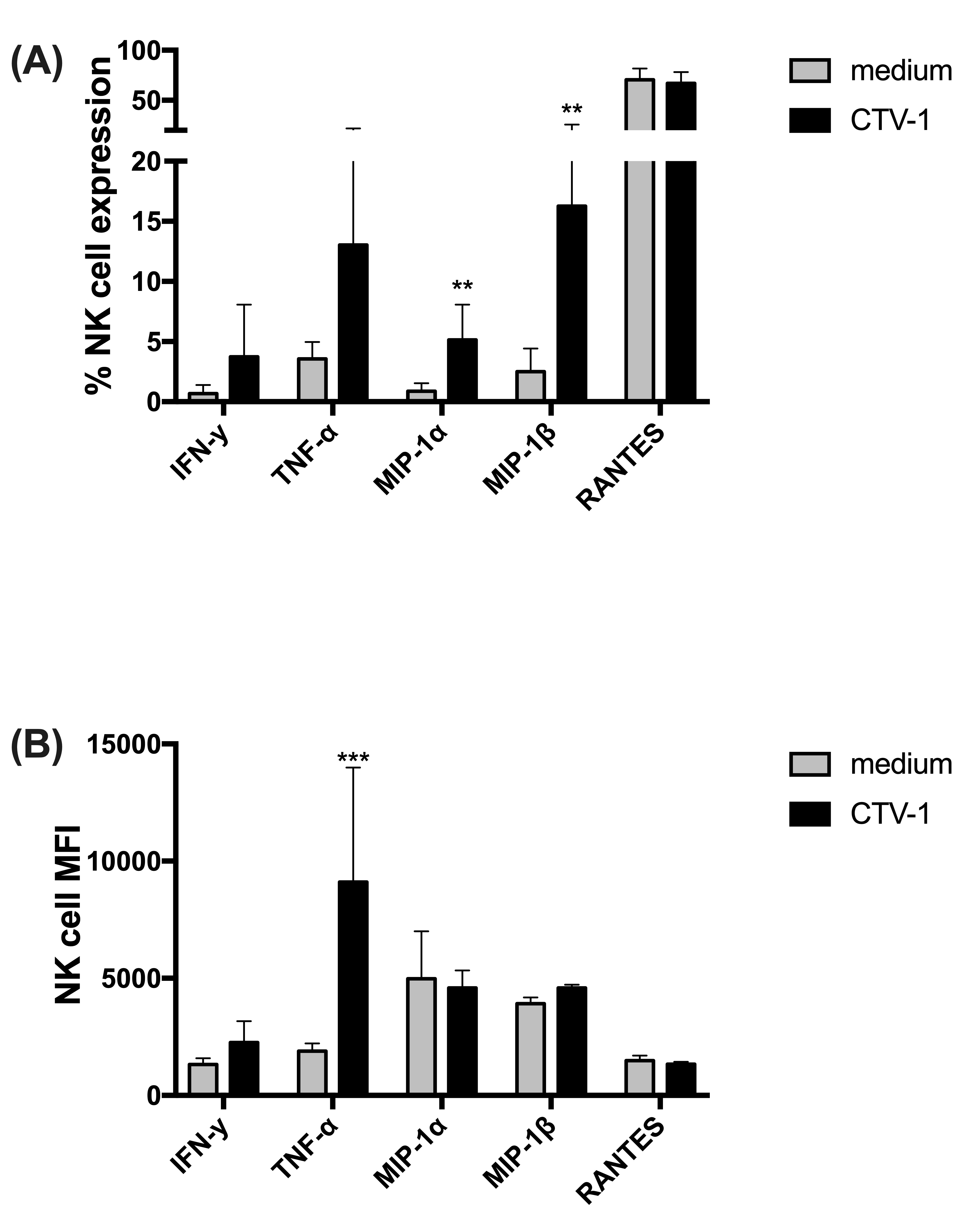

Supplement: S3 Fig — Freshly isolated NK cells were incubated in medium alone or with CTV-1 cells for 6 hours at 37°C. Then, cells were surface-stained with fluorochrome-conjugated anti-CD56 and anti-CD3 mAbs. Cells were fixed, permeabilized, and intracellularly stained with fluorochrome-conjugated mAbs against IFN-γ, TNF-α, MIP-1α, MIP-1β, and RANTES. Brefeldin A and/or monensin were added when appropriate 1 hour after incubation. Appropriate isotype and FMO controls were included in each experiment. (A) Bars represent mean percentage NK cell expression ± SD of 3–7 different donors. (B) Bars represent mean NK cell median fluorescence intensity values ± SD of 3 different donors. NK cell expression after stimulation was compared with cells treated with medium alone using the paired t-test. Statistical significance is indicated as: *P <0.05; **P <0.01; ***P <0.001. (TIF) [file pone.0218674.s003.tif]

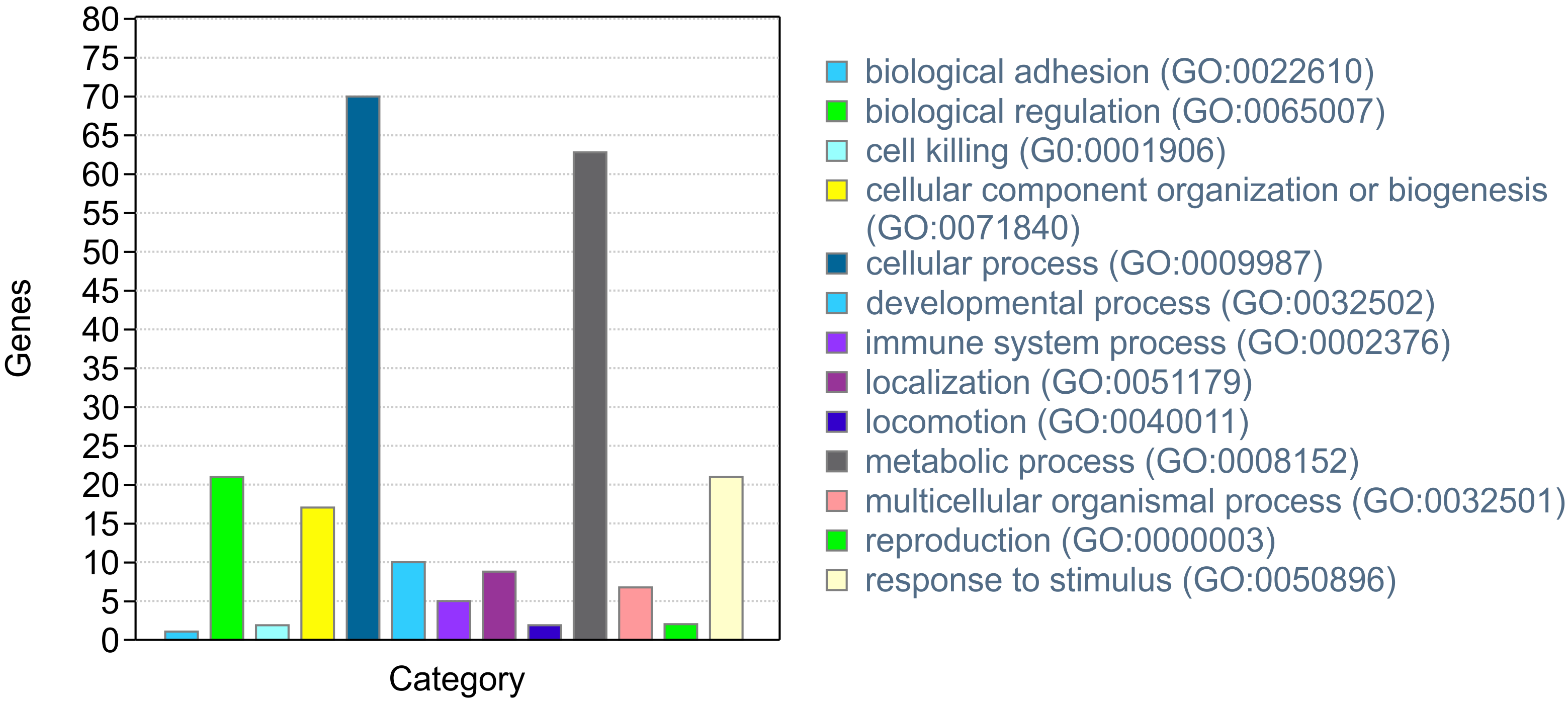

Supplement: S4 Fig — Freshly isolated NK cells were incubated in medium alone, with mitomycin-C- treated K562 or CTV-1 cells for 6 hours, or with IL-2 overnight. Then, NK cells were selectively enriched from the co-culture, mRNA was extracted, and gene expression was analyzed using RNA-sequencing. Functional annotation analysis using gene ontology terms was performed on genes commonly upregulated after exposure to K562, CTV-1, or IL-2 relative to NK cells incubated in medium alone that met the cutoff values (>1 Log2fold change, P-value <0.05) using the Panther Classification System gene list analysis tool. (TIF) [file pone.0218674.s004.tif]

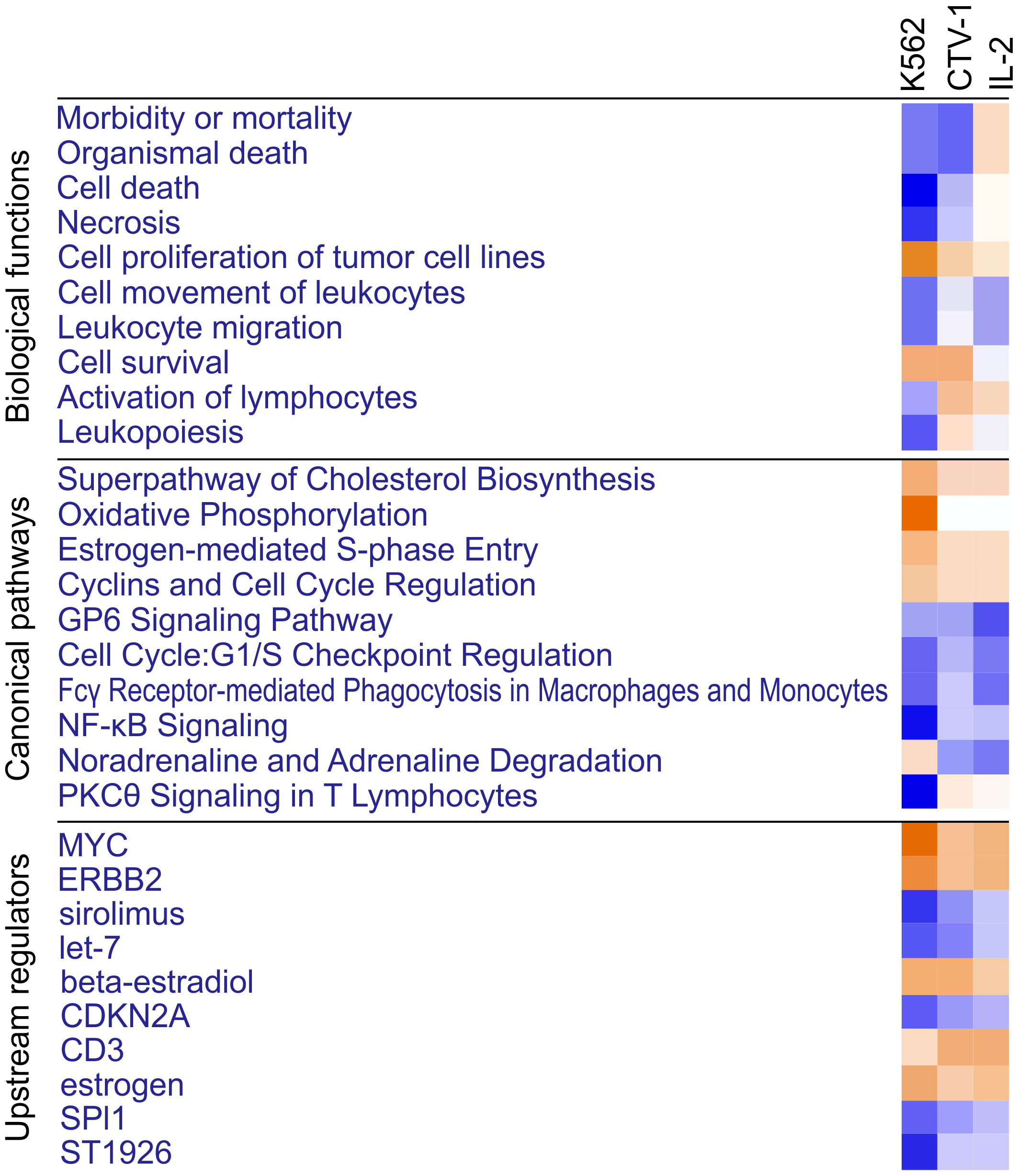

Supplement: S5 Fig — Freshly isolated NK cells were incubated in medium alone, with mitomycin-C-treated K562 or CTV-1 cells for 6 hours, or with IL-2 overnight. Then, NK cells were selectively enriched from the co-culture, mRNA was extracted, and gene expression was analyzed using RNA-sequencing. Biological functions, canonical pathways, and upstream regulators associated with NK cell genes variably expressed after exposure to K562, CTV-1, or IL-2 relative to NK cells incubated in medium alone that met the cutoff values (>1 Log2fold change, P-value <0.05) were analyzed using Ingenuity pathway analysis software. Colors indicate predicted activity according to activation z-scores, with orange predicting an overall increase and blue indicating a decrease in activity relative to NK cells incubated in medium alone. (TIF) [file pone.0218674.s005.tif]

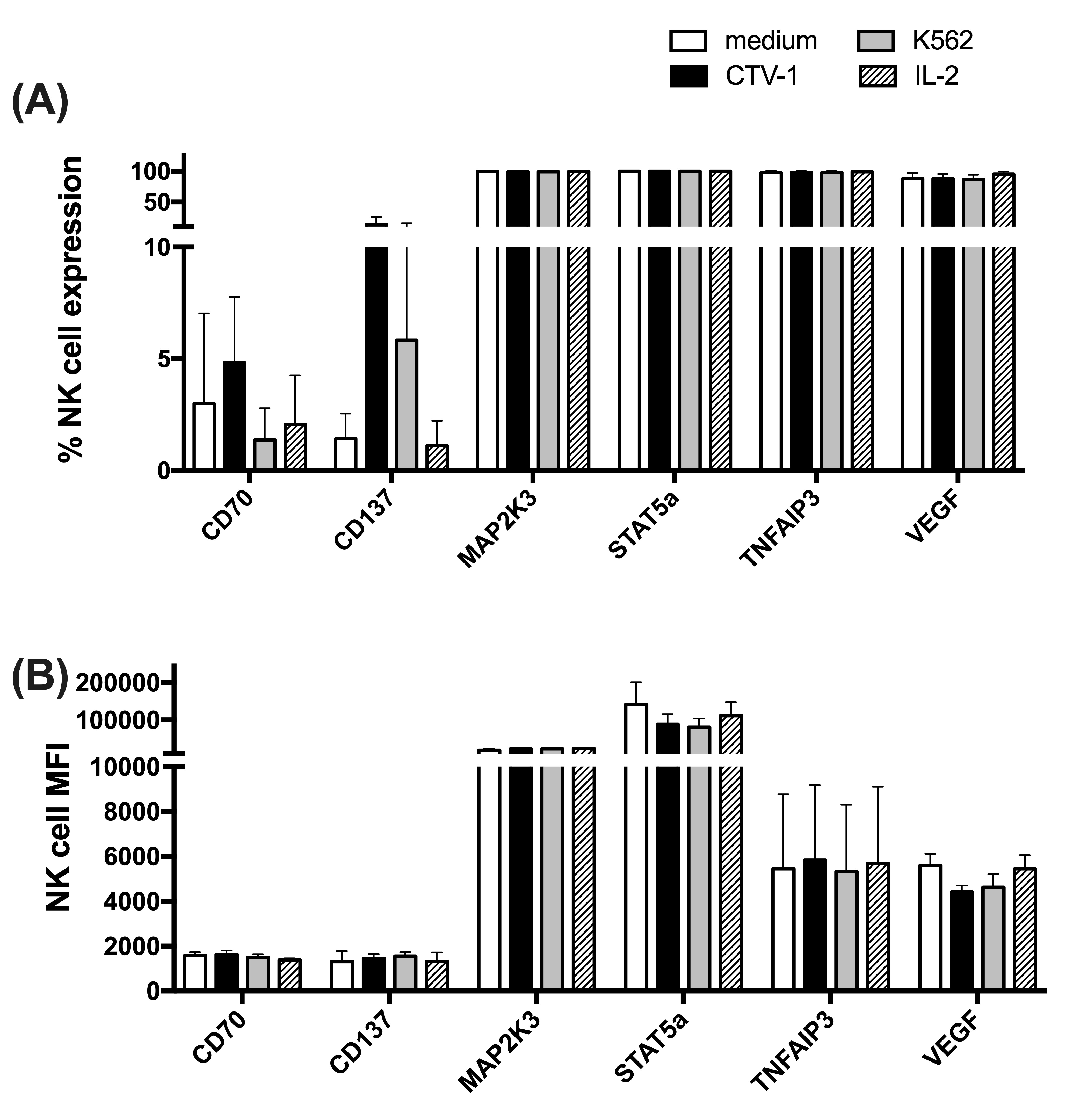

Supplement: S6 Fig — Freshly isolated NK cells were incubated in medium alone or with K562 or CTV-1 cells for 6 hours or IL-2 overnight at 37°C. Then, cells were surface stained with fluorochrome-conjugated anti-CD56, anti-CD3, anti-CD137, and anti-CD70 mAbs. Cells were fixed, permeabilized, and intracellularly stained with fluorochrome-conjugated mAbs against STAT5a, MAP2K3, TNFAIP3, and VEGF. Brefeldin A and/or monensin were added when appropriate 5 hours before the end of the incubation period. Appropriate isotype and FMO controls were included in each experiment. (A) Bars represent mean percentage NK cell expression ± SD of 3–5 different donors. (B) Bars represent mean NK cell median fluorescence intensity values ± SD of 3–5 different donors. (TIF) [file pone.0218674.s006.tif]
