# Supplementary material for: Tumor- and cytokine-primed human natural killer cells exhibit distinct phenotypic and transcriptional signatures
Source: PLoS One. 2019 Jun 26;14(6):e0218674. doi: 10.1371/journal.pone.0218674 (PMC6594622; doi:10.1371/journal.pone.0218674)
Supplement: S1 Table — (DOCX) [file pone.0218674.s007.docx]

| **Cell line** | HLA-ABC | MICA/  MICB | ULBP1 | ULBP3 | ULBP  2/5/6 | CD155 | CD112 | B7-H6 |
| --- | --- | --- | --- | --- | --- | --- | --- | --- |
| **K562** | -/+ | + | + | -/+ | -/+ | ++ | ++ | + |
| **CTV-1** | + | + | + | -/+ | + | + | + | + |
| **DAUDI** | -/+ | + | -/+ | - | - | -/+ | -/+ | -/+ |
| **RPMI-8226** | ++ | + | -/+ | -/+ | -/+ | ++ | + | + |
| **MCF-7** | ++ | + | -/+ | -/+ | -/+ | ++ | ++ | - |

# S1 Table. Expression of ligands for NK cell receptors on different tumor cell lines.

**The (-) symbol denotes absence of expression relative to the fluorescence minus one control, (+/-) indicates <5000, (+) 5000-50,000 and (++) >50,000 median fluorescence intensity values.**
