# Supplementary material for: Tumor- and cytokine-primed human natural killer cells exhibit distinct phenotypic and transcriptional signatures
Source: PLoS One. 2019 Jun 26;14(6):e0218674. doi: 10.1371/journal.pone.0218674 (PMC6594622; doi:10.1371/journal.pone.0218674)
Supplement: S2 Table — (DOCX) [file pone.0218674.s008.docx]

# S2 Table. Overlap in NK cell gene expression from RNA-sequencing analysis after stimulation with K562, CTV-1, or IL-2.

| **Change in expression** | **Genes affected (vs medium)** |
| --- | --- |
| Upregulation | *AMOTL1, ARG2, ASAH2B, ASF1B, AUNIP, BCL2L1, BCL6B, BEND3, C16orf46, C16orf59, CAMSAP3, CDC25A, CDC42EP2, CDC45, CDC6, CDCA5, CDCA7, CDT1, CENPM, CENPU, CHAC1, CHEK1, CKS2, CLSPN, CREM, CSRNP1, CTNNAL1, CTPS1, DDIAS, DHCR7, DONSON, DSCC1, DTL, DUSP5, E2F1, E2F2, EGR1, EGR3, ERCC6L, ESPL1, ETV4, EXO1, FOSB, GCAT, GINS1, GINS2, GMNN, GPT2, GRHL1, GSG2, GTPBP4, HAPLN3, HMGB3, HMGCS1, HOXB5, HOXB9, HSPA1A, HSPD1, IER5, IRX5, KCNH4, KCNK5, KIAA0101, KIAA1522, KIF18A, KPNA2, LMNB2, LRRC8B, MAD2L1, MCM6, MMD, MYBL2, MYRF, NDFIP2, NDUFA4L2, NOCT, NR4A1, NR4A2, NR4A3, OIP5, ORC1, ORC6, PHLDA1, PKMYT1, PLXNB1, PMAIP1, POLQ, PSAT1, PYCR1, RAD51, RAD51AP1, RAD54L, RBBP8, RRAD, SDF2L1, SHCBP1, SIGLEC12, SKA3, SLC16A1, SLC27A2, SLC6A9, SLCO4A1, SLFNL1-AS1, SNORA28, SNORD83A, SOCS1, SOCS2, SPAG4, SPRY4, SQLE, STAP2, STC2, SUV39H2, TK1, TRIP13, TUBB3, UBE2T, UHRF1, ULBP1, ULBP2, USP49, WDR62, WEE1, ZC3H12A* |
| Downregulation | *A2M, ABCA1, ACP5, AFF3, ALDH3B1, AMIGO1, BHLHE41, C3, CACNA1C-AS1, CAPN12, CD9, CDK14, CLMN, COL24A1, COL4A4, CTSK, CTSS, CYBB, CYBRD1, CYGB, DCSTAMP, DISP2, DTX4, EEPD1, ENG, FLJ37035, FLJ44635, FLRT2, FLVCR2, FMN1, FMO5, GM2A, HMOX1, HSD17B14, HTR7P1, IFNG-AS1, KCNMA1, KIAA0930, KRT2, KRT74, LDB2, LGALS2, LRP1, LRRC25, LRRN1, LTF, MARCH1, NFAM1, NLRC4, NR1H3, PACSIN1, PAPSS2, PCMTD2, PILRA, PRAM1, PSD3, RASAL1, RASGRP2, RGL1, RIN2, RPS6KA2, SH3RF3, SIRPB1, SLC29A3, SLC37A2, ST14, STARD13, TBC1D2, TBC1D9, TIMP2, TMEM204, TMEM255A, TMEM91, TNFSF13B, TNR, TPM2, TSPAN32, UNC45B, WDFY4, ZMAT4, ZMIZ1-AS1, ZNF471* |
