# Supplementary material for: Tumor- and cytokine-primed human natural killer cells exhibit distinct phenotypic and transcriptional signatures
Source: PLoS One. 2019 Jun 26;14(6):e0218674. doi: 10.1371/journal.pone.0218674 (PMC6594622; doi:10.1371/journal.pone.0218674)
Supplement: S3 Table — (DOCX) [file pone.0218674.s009.docx]

# S3 Table. Top 50 variably expressed NK cells genes according to log2fold change from RNA-sequencing analysis after NK cell exposure to K562 cells.

| Gene | Log2Fold Change (vs medium) |
| --- | --- |
| HSPA1A | +24.59 |
| RHAG | +15.23 |
| CTCFL | +15.11 |
| FLNC | +14.97 |
| HBG2 | +14.90 |
| HBE1 | +14.42 |
| IGF2BP1 | +14.37 |
| DLK1 | +14.34 |
| HBG1 | +14.19 |
| SSX1 | +14.10 |
| XIST | +14.07 |
| GABRE | +14.03 |
| NMU | +13.77 |
| KRT19 | +13.76 |
| MAGEB2 | +13.55 |
| GALNT5 | +13.49 |
| PCSK9 | +13.45 |
| LOC283352 | +13.44 |
| MAGEC2 | +13.44 |
| LINC01287 | +13.25 |
| NES | +13.19 |
| OR51B5 | +13.18 |
| MAGEA12 | +13.17 |
| GYPA | +13.07 |
| EVADR | +13.07 |
| SIGLEC1 | -8.90 |
| ITGAD | -7.92 |
| LINC01504 | -7.46 |
| OLFM4 | -6.98 |
| SULT1B1 | -6.93 |
| KGFLP2 | -6.86 |
| KLHL31 | -6.80 |
| KLHL35 | -6.78 |
| SIGLEC15 | -6.71 |
| A2M | -6.69 |
| PRR34 | -6.67 |
| UNC45B | -6.66 |
| LOC100996286 | -6.66 |
| TMEM171 | -6.64 |
| THNSL2 | -6.61 |
| GOLGA8J | -6.51 |
| RGMA | -6.48 |
| SLC47A1 | -6.47 |
| SDK2 | -6.39 |
| MIR181B2 | -6.36 |
| BHLHE41 | -6.34 |
| KRT73-AS1 | -6.25 |
| NSG1 | -6.21 |
| CCDC3 | -6.14 |
| ABCB5 | -6.14 |

# 
