# Supplementary material for: Tumor- and cytokine-primed human natural killer cells exhibit distinct phenotypic and transcriptional signatures
Source: PLoS One. 2019 Jun 26;14(6):e0218674. doi: 10.1371/journal.pone.0218674 (PMC6594622; doi:10.1371/journal.pone.0218674)
Supplement: S4 Table — (DOCX) [file pone.0218674.s010.docx]

# S4 Table. Top 50 variably expressed NK cells genes according to log2fold change from RNA-sequencing analysis after NK cell exposure to CTV-1 cells.

| Gene | Log2Fold Change (vs medium) |
| --- | --- |
| HSPA1A | +22.36 |
| PAGE5 | +9.76 |
| ASS1 | +9.49 |
| STC2 | +9.22 |
| LIN28B | +9.07 |
| ZIC2 | +8.77 |
| LOC728084 | +8.40 |
| PNMA2 | +8.14 |
| ABCC8 | +7.76 |
| LOC643201 | +7.64 |
| IRX5 | +7.60 |
| TUSC3 | +7.56 |
| WT1 | +7.44 |
| OGDHL | +7.14 |
| LINC00624 | +7.12 |
| GPR158 | +7.06 |
| PABPC4L | +7.04 |
| GDF10 | +6.98 |
| ASIC1 | +6.98 |
| DZIP1 | +6.95 |
| PITX1 | +6.94 |
| ETV4 | +6.94 |
| TMSB15A | +6.86 |
| TSPAN7 | +6.74 |
| NKAIN4 | +6.71 |
| SLCO2B1 | -7.63 |
| SIRPB2 | -7.32 |
| MS4A4A | -7.07 |
| DCSTAMP | -6.84 |
| CYP2S1 | -6.70 |
| FLRT2 | -6.50 |
| LYZ | -6.39 |
| ALDH1A1 | -6.39 |
| BHLHE41 | -6.38 |
| HNMT | -6.32 |
| NFAM1 | -6.18 |
| FAM198B | -6.18 |
| NUPR1 | -6.17 |
| FXYD6 | -6.01 |
| VNN1 | -5.97 |
| NLRC4 | -5.93 |
| LGALS2 | -5.89 |
| EPHB2 | -5.85 |
| PGM5 | -5.79 |
| RASAL1 | -5.69 |
| CYP27A1 | -5.56 |
| FPR3 | -5.40 |
| GPNMB | -5.38 |
| APOC1 | -5.35 |
| PPAP2B | -5.28 |
