# Supplementary material for: Tumor- and cytokine-primed human natural killer cells exhibit distinct phenotypic and transcriptional signatures
Source: PLoS One. 2019 Jun 26;14(6):e0218674. doi: 10.1371/journal.pone.0218674 (PMC6594622; doi:10.1371/journal.pone.0218674)
Supplement: S5 Table — (DOCX) [file pone.0218674.s011.docx]

# S5 Table. Top 50 variably expressed NK cells genes according to log2Fold change from RNA-sequencing analysis after NK cell exposure to IL-2.

| Gene | Log2Fold Change (vs medium) |
| --- | --- |
| HSPA1A | +18.63 |
| ETV4 | +6.49 |
| JPH2 | +5.96 |
| TBR1 | +5.60 |
| ARHGAP31-AS1 | +5.56 |
| NDUFA4L2 | +5.51 |
| CYP26A1 | +5.36 |
| LOC100133669 | +5.32 |
| SPRY4 | +5.30 |
| ADGRL2 | +5.20 |
| BCL6B | +5.12 |
| FAM27B | +5.07 |
| PTGER1 | +4.95 |
| MIR324 | +4.90 |
| C3orf52 | +4.64 |
| KCTD16 | +4.41 |
| STC2 | +4.26 |
| CDC45 | +4.22 |
| MIPOL1 | +3.98 |
| AS3MT | +3.88 |
| LIF | +3.87 |
| HBB | +3.84 |
| CDT1 | +3.83 |
| SOCS2 | +3.82 |
| IRX5 | +3.78 |
| FCN1 | -7.92 |
| VNN1 | -7.84 |
| MS4A7 | -7.41 |
| SIRPB2 | -7.34 |
| HNMT | -7.30 |
| ATP6V0D2 | -7.28 |
| APOC1 | -7.21 |
| MS4A4A | -7.09 |
| FAM198B | -7.01 |
| C16orf74 | -6.86 |
| MPO | -6.85 |
| NLRP12 | -6.80 |
| ALDH1A1 | -6.74 |
| LOC100506585 | -6.74 |
| MME | -6.62 |
| CSF3R | -6.61 |
| FMN1 | -6.42 |
| IFNG-AS1 | -6.40 |
| PTGES | -6.28 |
| NUPR1 | -6.18 |
| SLCO2B1 | -6.13 |
| COLEC12 | -6.08 |
| GPNMB | -6.02 |
| GLYCTK-AS1 | -6.01 |
| CPVL | -5.99 |
