# Supplementary material for: Tumor- and cytokine-primed human natural killer cells exhibit distinct phenotypic and transcriptional signatures
Source: PLoS One. 2019 Jun 26;14(6):e0218674. doi: 10.1371/journal.pone.0218674 (PMC6594622; doi:10.1371/journal.pone.0218674)
Supplement: S6 Table — (DOCX) [file pone.0218674.s012.docx]

**S6 Table. Tumour-induced changes from RNA-sequencing analysis in NK cell gene expression after NK cell exposure to K562 or CTV-1.**

| **Change in expression** | **Genes affected (vs medium)** |
| --- | --- |
| Upregulation | *ACAT2, ACBD7, ADSSL1, AEN, ALAS2, ALDH1A2, ANGPTL4, ANKLE1, ANKRD37, ANKRD9, ANTXR1, AOC2, AREG, ARHGEF17, ASIC1, ASS1, ATP2A1, ATP7B, AURKA, AURKB, BAG3, BAMBI, BEND6, BEX1, BEX2, BNIP1, BRE-AS1, BTG3, C10orf35, C11orf96, C17orf53, C19orf48, C19orf81, C1orf116, C6orf132, CALB1, CATSPERD, CBARP, CBX2, CCDC113, CCNB1, CCNB2, CCNO, CD55, CDCA8, CDR2, CDR2L, CENPV, CENPW, CFAP45, CKB, COCH, COL1A1, COL1A2, CPXM1, CRNDE, CSAG1, CTCFL, CTH, CTSV, CTU2, CTXN1, CXCL11, DDIT4, DLK1, DLL4, DLX1, DLX2, DMD, DNAAF3, DNAJB1, DNAJB5, DNAJC12, DNM1, DNMT3B, DNPH1, DOCK6, DRP2, DXO, E2F8, EFCAB12, EHD2, ELL2, ERRFI1, ESCO2, ETS2, ETV5, EZH2, FAM131A, FAM171A2, FAM46C, FAM83A, FANCI, FDXR, FERMT2, FGFR3, FKBP14, FLNC, FN1, FNDC4, FNDC5, FOXM1, FSCN1, FUT3, FZD5, GABRE, GAD1, GADD45A, GAGE1, GALNT5, GATA1, GATA2, GBAT2, GINS4, GPR158, GRIN2C, GXYLT2, GYPA, HBA1, HBE1, HBG1, HBG2, HEPH, HES1, HES4, HEY1, HIST1H1D, HIST1H2AE, HIST1H2BJ, HIST1H3B, HIST1H4I, HIST2H2BF, HMGA1, HMX3, HOXB6, HOXB8, HOXC9, HSP90AA1, HSPA4L, HSPA6, HSPH1, HYKK, ICAM5, ID1, IFRD1, IGF2BP1, IL15RA, ING2, JARID2-AS1, JUNB, KCND1, KCNH2, KIF18B, KIF2C, KREMEN2, KRT19, KRT8, LIN28B, LINC00624, LINC00885, LINC00958, LINC01021, LINC01029, LINC01287, LINC01419, LMNB1, LOC100506844, LOC102723828, LOC102724279, LOC283352, LOC643201, LOC728084, LSMEM1, MAFF, MAGEA12, MAGEA3, MAGEB1, MAGEC1, MAP1A, MAP1B, MARCKSL1, MASTL, MBOAT2, MCAM, MECOM, METTL21A, MEX3A, MFGE8, MKRN3, MND1, MNX1, MSMO1, MSX1, MTSS1L, MYB, MYEOV, MYLK3, NAB2, NAMPT, NANOS1, NAT8L, NET1, NFIB, NFIL3, NFKBIE, NKPD1, NLGN2, NMU, NPAS1, NR2F2, NR2F2-AS1, NT5C3B, NTRK1, NUSAP1, NXT1, OGDHL, ONECUT2, OR51B5, OSR2, OTX1, OVGP1, P3H3, PABPC4L, PACSIN3, PAGE5, PANX2, PAQR3, PAQR6, PARD3, PBK, PCAT6, PCSK9, PDCD2L, PDE4C, PELI1, PGF, PHGDH, PHLDB1, PIM3, PITX1, PLEKHH3, PLK4, POLB, POMGNT2, POTEE, PPP1R15A, PRC1, PRSS57, PTGER3, PTP4A1, PTPRS, PTRF, PTTG1, RASD1, RELT, RFPL4B, RHAG, RHBDL1, RNA45S5, RND1, RNF182, RNU86, ROBO2, SAGE1, SAMSN1, SCART1, SELENBP1, SEMA3F, SIX1, SIX4, SLC22A4, SLC22A5, SLC25A21, SLC25A33, SLC35G2, SLC45A3, SLC6A8, SLC9A5, SMTN, SNAI1, SNAPC1, SNHG15, SNHG16, SNORA40, SNORA45B, SNORA52, SNORD14C, SNORD16, SNORD25, SNORD68, SNORD99, SP6, SPAG5, SPATA2, SPDYC, SPTBN2, SSX1, SSX3, ST8SIA6-AS1, STARD10, STARD4, STAT5A, STMN1, STON2, STRA6, SUV420H2, TEAD2, TFAP2A, TICRR, TMEM231, TMEM56, TMEM97, TMSB15A, TNFRSF10B, TNNI3, TNNT1, TP53INP2, TRAF4, TRIM45, TRIM7, TSPAN13, TUBB2A, TUFT1, TUSC3, VANGL2, VGF, WDR34, WNK4, WT1, WT1-AS, WWC2, XIRP1, XIST, YES1, ZBTB21, ZIC2, ZIC5, ZNF730, ZUFSP* |
| Downregulation | *ACTA2-AS1, ADNP-AS1, AFAP1L2, AGAP1, AHNAK, AHRR, ANXA4, APP, ARFGEF3, ARHGAP12, ARL10, ASCL2, AVIL, B3GALNT1, B4GALNT3, BANK1, BCL9L, BOK, C19orf35, C1orf145, C1orf162, C20orf197, C4orf47, C9orf139, CACNA2D2, CALHM1, CALHM2, CCR6, CNR2, CPEB2, CPM, CRIM1, CXCR1, CXCR2, CXXC4, DOCK1, DOK7, DYRK2, DYSF, EMBP1, ENPP1, EPHA4, ERBB2, ERP27, FAM111B, FGD4, FGFBP2, FMNL2, FMO4, FUT7, GAS1, GCNT1, GIMAP1, GIMAP5, GIMAP6, GIMAP8, GSN, HDAC9, HOXA3, HPCAL4, HS3ST3B1, HTRA4, HVCN1, IGLL5, IL10RB-AS1, ITGAD, JAKMIP2, KCNQ5, KCTD12, KIAA1324, KIT, KL, KLF8, LCN12, LCNL1, LILRB4, LIME1, LINC00469, LINC00908, LINC00921, LINC01504, LINGO2, LINGO3, LOC100506472, LOC101928020, LOC101929698, LOC102723703, LOC399715, LPAR5, LXN, MAN1C1, MBP, MFSD6L, MIRLET7BHG, MMP8, MMRN1, MORC2-AS1, MPZL2, MREG, MYO1F, NCAM1, NCEH1, NEK6, NLRC3, NOL4L, NOTCH1, NREP, NRP1, PAN3-AS1, PCSK5, PDE6G, PDGFD, PECAM1, PFKP, PIK3R6, PITRM1-AS1, PKI55, PLEKHG3, PLEKHO2, PLXDC2, PLXNA4, PLXNC1, PODN, PPFIA4, PPFIBP2, PPP3CB-AS1, PRMT2, PRR34-AS1, PRSS36, PTGDS, PTPRCAP, PTPRN2, PYGM, RAB36, RAB38, RASSF1, RASSF3, RCBTB2, RIMKLA, RNF13, ROR1, RUNX2, S100A9, S1PR4, SAMHD1, SDK2, SERP2, SGK223, SGSM1, SH3BP2, SH3BP5, SH3D21, SIGIRR, SIGLEC1, SIGLEC15, SIGLEC17P, SIGLEC9, SLAMF8, SLC16A7, SLC23A3, SLC25A53, SLC26A11, SLC2A13, SLC2A5, SLC4A10, SLC8A3, SLCO4C1, SNX18, SNX30, SPSB1, SPTSSB, STK38, STOM, STYK1, SULT1B1, SYK, SYNGR1, TBC1D10C, TBXAS1, TKTL1, TLR9, TMC8, TMEM163, TMEM169, TNFAIP8L2, TNFRSF10D, TNRC6C-AS1, TRAF3IP3, TRG-AS1, TRPM2, TSHZ3, TSPAN14, TTC22, TUBA8, UBTF, UCP3, UNC93B1, VIT, WDR81, WNT11, ZMYND10, ZNF185, ZNF234, ZNF594, ZNF792* |
