# Supplementary material for: Tumor- and cytokine-primed human natural killer cells exhibit distinct phenotypic and transcriptional signatures
Source: PLoS One. 2019 Jun 26;14(6):e0218674. doi: 10.1371/journal.pone.0218674 (PMC6594622; doi:10.1371/journal.pone.0218674)
Supplement: S7 Table — (DOCX) [file pone.0218674.s013.docx]

# S7 Table. Overlap in NK cell gene expression from NanoString nCounter ™ gene expression profiling after stimulation with CTV-1 or IL-2.

| **Change in expression** | **Genes affected (vs medium)** |
| --- | --- |
| Upregulation | *ADAM19, ADGRE5, ATF3, BATF3, BCL2, BIRC3 ,BTG2, CCL3, CCL4 ,CCL8, CD69, CD80, CD83, CDKN1A, CLIC4, CREM ,CSF2, CXCL11, CXCL9, FAS, FASN, FOSL1, FSCN1, GADD45B, HAVCR2, HPRT1, ICAM1, IDO1, IFNG, IL1A, IL1B, IL23A, IL4R, IRF4, IRF8, LIF, LTA, MAPK11, MIF, MYC, NAMPT, NFIL3, NFKB1, NFKBIA, NFKBIZ, PIM2, RGS1, SERPINB9, SOCS1, SOCS2, SOCS3, TNF, TNFRSF9, TNFSF10, TRAF, TUBA4A, TUBB, TXN* |
| Downregulation | *ALOX5, CLEC5A, CYBB, FBP1, FCN1, FPR3, GRN, GSN, LGALS3, LIPA, MPEG1, S100A8, S100A9, SIRPA, TLR6* |
